# Targeting STAT3-mediated lipid metabolism reprogramming overcomes chemoresistance in acute myeloid leukemia

**DOI:** 10.1038/s41419-026-08988-4

**Published:** 2026-06-17

**Authors:** Keren Peng, Jianshan Mo, Zhenjiao Yang, Wen Ding, Jiayu Yan, Shumin Ouyang, Minyuan Lu, Kai Zhu, Hongru Yao, Huiqin Chen, Xiongjun Xu, Peibin Yue, Jinjian Lu, Yuanxiang Wang, Shanyi Zhang, Yandong Wang, Xiaolei Zhang

**Affiliations:** 1 National-Local Joint Engineering Laboratory of Druggability and New Drug Evaluation, State Key Laboratory of Anti-Infective Drug Discovery and Development, Guangdong Key Laboratory of Chiral Molecule and Drug Discovery, School of Pharmaceutical Sciences, Sun Yat-sen University, Guangzhou, China; 2 State Key Laboratory of Ophthalmology, Zhongshan Ophthalmic Center, Sun Yat-sen University, Guangzhou, China; 3 Innovation Practice Center, Changchun University of Chinese Medicine, Changchun, China; 4 Department of Neurosurgery, Guangdong Provincial Key Laboratory of Malignant Tumor Epigenetics and Gene Regulation, Sun Yat-Sen Memorial Hospital, Sun Yat-Sen University, Guangzhou, China; 5 Department of Pediatrics, The Third Affiliated Hospital of Sun Yat-sen University, Guangzhou, China; 6 Department of Medicine, Division of Hematology-Oncology, and Samuel Oschin Comprehensive Cancer Institute, Cedars-Sinai Medical Center, Los Angeles, CA USA; 7 State Key Laboratory of Quality Research in Chinese Medicine, Institute of Chinese Medical Sciences, University of Macau, Macao, China

**Keywords:** Acute myeloid leukaemia, Targeted therapies

## Abstract

Chemotherapy resistance and intolerance present significant challenges in the effective treatment of acute myeloid leukemia (AML). However, the role of metabolic reprogramming, particularly lipid metabolic rewiring, in promoting chemotherapy resistance in leukemia has not been fully elucidated. Here, we found that multiple lipid metabolism processes are aberrantly activated in Ara-C resistant AML cells, accompanied by upregulation of JAK-STAT3 signaling and key lipid metabolic regulators, notably SREBP1 and CPT2. Additionally, we discovered W1307, a potent and highly selective STAT3 inhibitor, which demonstrated significant anti-tumor activity both in vitro and in vivo. Genetic and pharmacological inhibition of STAT3 simultaneously suppresses lipid synthesis and catabolism, leading to lipids metabolic disorder accompanied with lipids accumulation, ROS increase, lipid peroxidation and mitochondrial membrane potential decrease. Mechanistically, STAT3 binds to DNA response elements in the promoters of the lipid metabolism associated gene SREBF1 and CPT2, and regulates their expression. Furthermore, inhibition of STAT3 enhances the anti-tumor effect of Ara-C and sensitizes resistant AML cell line to Ara-C through disrupting lipid homeostasis and triggering lipotoxicity. Our findings highlight the critical role of STAT3-driven lipid metabolism reprogramming in chemoresistance. Furthermore, W1307 emerges as a promising therapeutic candidate to overcome chemoresistance in leukemia treatment.

**Figure:**
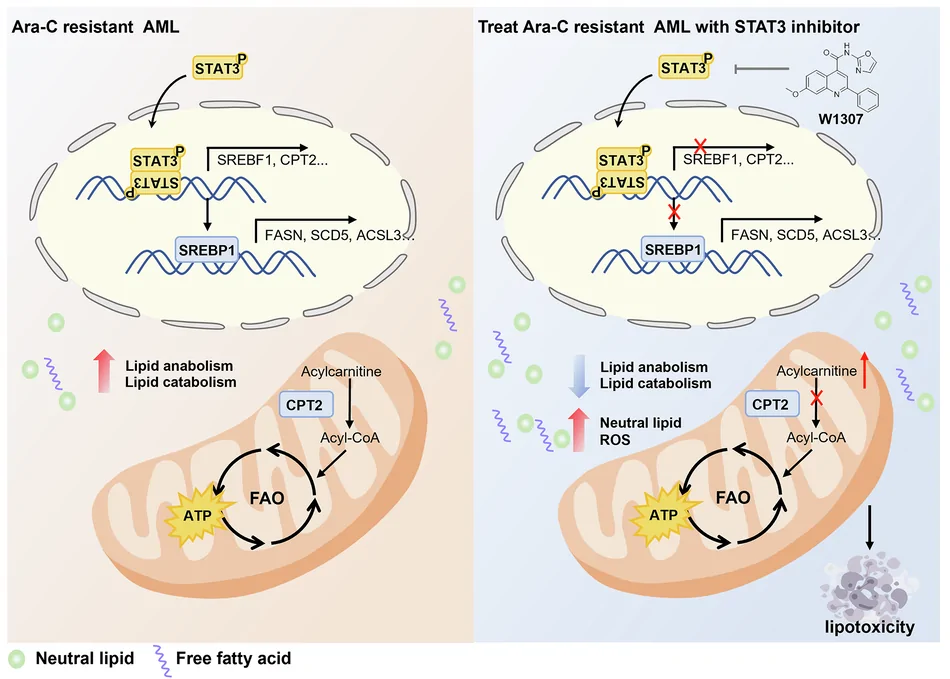

## Introduction

AML, a malignancy of the hematopoietic system, arises from aberrant myeloid cell expansion, resulting in bone marrow infiltration and subsequent damage to other tissues. Clinical manifestations include bruising, fever, organomegaly, and other debilitating symptoms [1, 2]. Despite decades of research, the therapeutic landscape for AML has seen limited evolution. Chemotherapy remains a first-line treatment for AML, with the standard “7 + 3” regimen combining 7 days of Ara-C and 3 days of anthracycline administration. As an anti-metabolic agent, the active form of Ara-C can disrupt DNA synthesis and block the cell cycle [3]. However, poor prognosis is still disquieting in AML patients, due to the emergence of drug resistance [4, 5]. Chemoresistance represents a major bottleneck for effective therapy in AML. In addition, Chemotherapy showed limited efficacy in patients over 60 [4]. Therefore, there is an urgent need for efficacious therapies to address advanced AML and chemoresistance.

Metabolism reprogramming, a hallmark of cancer cells, is crucial to provide enough energy and sustain macromolecules demand for cell growth and survival [6]. As key structural elements of cell membranes, lipids contribute significantly to cellular energy metabolism and tumorigenesis [7, 8]. Lipid metabolic reprogramming supports tumorigenesis and treatment resistance, involving alterations in biosynthesis, uptake, storage, and catabolism across various malignancies [9–12]. Additionally, the role of membrane lipid composition, as specified by double bond contents, in drug resistance has garnered increased recognition [13, 14]. Various oncogenic signals differentially regulate specific lipid metabolic pathways. For example, the transcriptional activation of SREBP1, a key lipogenesis-related transcription factor, is regulated by PI3K–AKT–mTORC1 axis [15]. The inhibition of mTORC1 has been demonstrated to down-regulate the level of critical enzymes, notably FASN, SCD, and ACC1, leading to diminished tumor growth [16]. Despite concerted efforts to unravel the regulatory crosstalk between lipid metabolism and oncogenic signaling, comprehensive mechanisms and contributions of their complex interplay to cancer progression and drug resistance remain elusive. Therefore, there is a compelling need for further investigation into the precise mechanisms for regulatory crosstalk between lipid metabolism and oncogenic signaling.

STAT3, known as a oncogenic transcriptional regulator, exhibits aberrant activation across various solid tumors and hematological malignancies [17–19], and significantly contributes to tumor aggression, metastasis, and promotes drug-resistance [20–22]. Growing evidence have demonstrated that STAT3 is crucial for leukemia progression and is hyperactivated in AML patients [23, 24]. Notably, STAT3 also significantly influences metabolic pathways. STAT3 could mediate OXPHOS in leukemia stem cells (LSCs), which population contributes to relapse and poor survival [24]. Further study showed that IL-6/STAT3 promotes fatty acid uptake through upregulating CD36 expression, leading to chemoresistance [25]. Considering the integral role of lipid metabolism in chemoresistance and functions of STAT3 [26], we supposed that STAT3 inhibition offers a promising approach to reverse AML chemoresistance through intervention in lipid metabolism.

Our findings identify upregulated lipid metabolism as associated with chemoresistance in AML. Mechanistically, STAT3 directly regulates SREBP1 and CPT2 expression through promoter binding. Both genetic and pharmacological STAT3 inhibition result in suppression of lipid synthesis and catabolism, resulting in lipid metabolic dysregulation, lipid accumulation, oxidative stress, lipid peroxidation, and mitochondrial failure. In view of these findings, we developed a selective STAT3 inhibitor W1307, which exhibits significantly anti-tumor effects. Importantly, the combination of W1307 with Ara-C restores chemosensitivity in resistant AML cells, inducing significant tumor regression in a resistant AML model.

## Materials and Methods

### Cell culture

The MOLM-13, K562, 5637, and HEK-293T cell lines were obtained from the American Type Culture Collection (ATCC, USA). MOLM-13 and K562 cells were cultured in RPMI-1640 (Gibco, Carlsbad, CA, USA) with 10% fetal bovine serum (FBS, Gibco, Carlsbad, CA, USA) and 1% penicillin-streptomycin (P.S., Gibco, USA). HEK-293T cells were cultured in DMEM (Gibco, USA) with 10% FBS and 1% P.S. Primary human leukemia cells were isolated and maintained in RPMI-1640 medium supplemented with 20% FBS, 20% culture supernatant from 5637 cells, and 1% ITS solution (Thermo Fisher). Ara-C-resistant MOLM-13/AR cells were generated through continuous exposure to a low dose of Ara-C, exhibiting a resistance index of 19.84. All cell lines were incubated in a 37 °C environment with 5% CO_2_. Cell line authentication was performed using STR analysis with the GenePrint 10 System (B9510, Promega, USA) following the manufacturer’s instructions.

### Primary human AML samples

Primary human AML samples were collected at the Third Affiliated Hospital of Sun Yat-Sen University (Guangzhou, China), with informed consent provided by all patients and under protocols strictly approved by the institutional Human Ethics Committee.

### Antibodies

Anti-β-actin (#sc-47778), Anti-Bcl-xL (#sc-8392), Anti-Bcl-2 (#sc-7382) were purchased from Santa Cruz (USA). Anti-STAT3 (#10253-2-AP), Anti-STAT1 (#10144-2-AP), Anti-Flag-tag (#66008-3-Ig), Anti-CPT1A (#15184-1-AP), Anti-CPT2 (#26555-1-AP), Anti-SREBF1 (#14088-1-AP), Anti-FASN (#10624-2-AP), Anti-ACSL3 (#20710-1-AP) were purchased from Proteintech (China). Anti-Phospho-STAT3 (Tyr705) (#9145), Anti-c-Myc (#5605), Anti-Mcl-1 (#4572), Anti-PhosphoSTAT1 (Tyr701) (#9167), Anti-Phospho-STAT5 (Tyr694) (#9351), Anti-STAT5 (#25656), Anti-AKT (#4685), Anti-Phospho-AKT (Ser473) (#4060, Anti-HA-tag (#3724), Anti-PARP (#9542), Anti-Cleaved Caspase-7 (#9491) were purchased from Cell Signaling Technology (USA). Anti-SCD5(#A13127) was purchased from ABclonal (China).

### Surface Plasmon Resonance (SPR) assay

The binding affinity between W1307 and STAT3 was analyzed using a Biacore 8 K instrument. Briefly, a purified truncated STAT3 protein (amino acids 127-722, MW: 68.7 kDa) was immobilized onto a CM5 chip (GE Healthcare, USA). The experiments were performed using a filtered running buffer consisting of 1× PBS, 0.05% surfactant P20, and 2% DMSO. To evaluate binding kinetics, a two-fold serial dilution of W1307 (12.5, 6.25, 3.12, 1.56, and 0.78 μM) was prepared in the running buffer and sequentially flowed over the immobilized chip surface to generate response signals. All kinetic parameters and the final binding affinity (KD) were processed and calculated using the Biacore Insight Evaluation software.

### Measurements of cholesterol and triglyceride levels

Cholesterol Quantification Kit (A111-1-1, Nanjing Jiancheng Bioengineering Institute, Nanjing, China) and Triglyceride Colorimetric Assay Kit (A110-1-1, Nanjing Jiancheng Bioengineering Institute, Nanjing, China) were used following the manufacturer’s instructions.

### Molecular docking

Molecular docking simulations were performed utilizing the Schrödinger software suite (Maestro version 11.1). The crystal structure of the STAT3 protein (PDB ID: 6NUQ) was retrieved from the Protein Data Bank (http://www.pdb.org). Prior to docking, the receptor structure was refined using Schrödinger’s Protein Preparation Wizard to assign bond orders, add missing hydrogen atoms, and optimize the hydrogen bond network. Concurrently, the 3D structures of the small molecules were optimized, and their appropriate ionization states were generated using Schrödinger’s LigPrep module.

To define the binding pocket, the active site grid box was generated using the Receptor Grid Generation tool, with the grid coordinates strictly centered around the SH2 domain of STAT3. The molecular docking was executed using the “Ligand Docking” module. During the docking process, protein was considered rigid, and small molecules were flexible during docking process. Crucially, the Extra Precision (XP) scoring function was selected to accurately evaluate and rank the binding poses. Finally, the electrostatic characteristics of the binding interfaces were visualized using Schrödinger’s Protein Surface Analyzer, with the receptor surface color-coded based on electronegativity to map the molecular interactions.

### CETSA

K562 cells (15 cm dishes) were treated with W1307 or DMSO for 1 h. After PBS washing, cells were resuspended in 1 mL PBS containing the corresponding compounds. The suspension was aliquoted into 7 PCR tubes, heated for 2 min at various temperatures. Samples were incubated for 3 min at room temperature. 3 freeze-thaw cycles (liquid N₂-37 °C water bath) were performed to disrupt cells. Lysates were centrifuged (20,000 × g, 20 min, 4 °C), and supernatants were mixed with loading buffer for Western blotting.

### Luciferase reporter assays

HEK-293T cells in 96-well plates were co-transfected with plasmids (50 ng pGL3-STAT3, 50 ng STAT3C, and 40 ng TKRL) using Lipofectamine 2000, followed by 24 h W1307 treatment at varying concentrations. Luciferase activity was assessed using the Dual-Luciferase Reporter Assay Kit (Beyotime).

### Co-Immunoprecipitation (CO-IP)

HEK-293T cells were co-transfected with HA-STAT3 and Flag-STAT3 plasmids, treated with W1307 (24 h), and stimulated with IL-6 (1 h). Following lysis in IP RIPA buffer, Flag-STAT3 was immunoprecipitated overnight at 4 °C using anti-Flag beads. After PBST washing, proteins were eluted with loading buffer and analyzed by Western blot.

### CRISPR/Cas9-mediated STAT3 knockout

An exon 2-targeting STAT3 guide RNA was cloned into lentiCRISPRv2 to generate sgSTAT3, which was then delivered into MOLM-13 cells via lentiviral transduction followed by puromycin selection. The gRNA used for STAT3 knockout is listed in Suppl. Table S1.

### Cell viability assays

Cell viability was assessed by CCK-8 assay (Selleck, USA). Leukemia cells (1 × 10^4^ cells/well) were seeded in 96-well plates, treated with drug gradients for 72 h, then incubated with 10 μL of CCK8 solution for 1–4 h at 37 °C. Absorbance at 450 nm was measured using a microplate reader (FLUOstar Omega-ACU, USA).

### EDU assay

The EDU assay was performed using the BeyoClick™ EdU Cell Proliferation Kit with Alexa Fluor 555 (C0075S, Beyotime, Shanghai, China). Cell proliferation was assessed by flow cytometry.

### Cell apoptosis assay

Cell apoptosis was assessed using the Annexin V-FITC Apoptosis Detection Kit I (BB-4101, Bestbio, Shanghai, China). Apoptotic cells were detected by flow cytometry.

### ROS assay

Total ROS levels were measured using the ROS Assay Kit (S0033, Beyotime, Shanghai, China). DCFH-DA (10 μM in serum-free medium) was used to probe cellular ROS levels by incubating with treated/untreated cells at 37 °C for 30 min. The ROS levels were measured by flow cytometry.

### Lipid ROS and MDA assay

Lipid ROS levels were detected with C11-BODIPY 581/591 (Invitrogen, Carlsbad, CA, USA) followed by flow cytometric analysis. MDA contents were determined using the Lipid Peroxidation MDA Assay Kit (S0131S, Beyotime, Shanghai, China).

### Detection of neutral lipid contents by BODIPY 493/503 assay

Neutral lipid content was detected using BODIPY 493/503 (HY-W090090, MCE, Matawan, NJ, USA). The probe was diluted to a concentration of 10 μM. After 20 min incubation at 37 °C and washing, lipid content was quantified by flow cytometry.

### RT-PCR

Total RNA was extracted with TRIeasy™ reagent (10606ES60, Shanghai, China) and reverse transcribed into complementary DNA (cDNA) using the Hifair™ II 1st Strand cDNA Synthesis SuperMix Kit (11123ES60, YEASEN, Shanghai, China). Quantitative real-time PCR was performed using a BIO-RAD CFX96™ system (Bio-Rad, San Diego, USA) with SYBR Green (11201ES08*, YEASEN, Shanghai, China). Primer sequences used in this study are listed in Suppl. Table S2.

### Measurement of Fatty Acid Oxidation (FAO) Rate

The cellular fatty acid oxidation rate was quantified using a commercial FAO Assay Kit (BC0815, Solarbio Science & Technology, Beijing, China) in accordance with the manufacturer’s instructions. Briefly, approximately 5 × 10^6^ cells were harvested, washed, and resuspended in 1 mL of the provided extraction buffer. The cells were then mechanically disrupted via sonication on ice. Following centrifugation at 10,000 g for 10 min at 4 °C, the supernatant was collected. For the assay, 50 μL of the sample supernatant was mixed with the working solutions prepared from the kit components in a 96-well plate. The assay measures the production of NADH during fatty acid β-oxidation, which reacts with a color developer to form an orange-red complex. After a 30 min incubation at 37 °C, the absorbance was measured at 485 nm using a microplate reader. The FAO rate was calculated based on a standard curve generated with NADH standards.

### Chip-qPCR analysis

MOLM-13/AR cells exposed to W1307/vehicle for 48 h. ChIP-qPCR assay was performed as previously described [27]. The primers are listed in Supplemental Table S3.

### Western blot analysis

Cell and tissue lysates were prepared using RIPA buffer (P0013B, Beyotime, Shanghai, China) containing protease inhibitors (B14001, Selleck, USA) and phosphatase inhibitors (B15001, Selleck, USA). Protein quantification was performed using Pierce™ BCA Protein Assay Kit (23225, Thermo, USA). Proteins were separated by 8–15% SDS-PAGE and transferred to PVDF membranes (Millipore, USA). Following blocking, membranes were probed with primary antibodies (4 °C, overnight) and secondary antibodies, with detection performed using the Bio-Rad ChemiDoc MP system (1708280, Bio-Rad, USA) after incubation with ECL solution.

### Bioinformatics analysis

Gene Set Enrichment Analysis (GSEA) was performed using GSEA desktop software. Pathway enrichment analysis was conducted using the online GSEA tool (https://www.gsea-msigdb.org/gsea). Clinical survival analysis and gene correlation were assessed using the GEPIA online database, a web-based platform for genomic analysis and visualization.

### RNA sequencing

For RNA sequencing, cell samples were sent to Sangon Biotech (Shanghai, China) for RNA extraction, library preparation, and high-throughput sequencing. Quality control of the sequenced data was performed using FastQC, and HTSeq was employed to align cleaned reads to the reference genome. Differential expression analysis was conducted using DESeq2.

### Lipidomics

Cell samples were sent to Metware (Wuhan, China) for lipid metabolite extraction and analysis. Lipid metabolites were extracted from cells using a lipid extraction solution with internal standards. The supernatant was collected after sonication, vortexing, and centrifugation. UPLC-MS/MS analysis was performed using an ExionLC™ AD system (https://sciex.com.cn/) and a QTRAP® 6500+ mass spectrometer (https://sciex.com.cn/). Lipid contents were analyzed using the data from mass spectrometry and the Metware Database (MWDB). Quantification was performed using the multiple reaction monitoring (MRM) mode of the triple quadrupole mass spectrometer.

### Plasmid construction, cell transfection, and virus infection

Stable gene knockdown was performed using lentiviral-based shRNA, while transient gene knockdown was achieved using small interfering RNAs (siRNAs). The sequences of shRNAs and siRNA are listed in Suppl. Table S4. For gene overexpression, transient plasmid transfection was utilized. Specifically, the overexpression plasmids for SREBF1 (NM_004176.5), pLV3-CMV-SREBF1(human)-3×FLAG-CopGFP-Puro, and CPT2 (NM_000098.3), pLV3-CMV-CPT2(human)-3×FLAG-CopGFP-Puro, were purchased from MiaoLing Biology (P63810 and P69659, Wuhan, China). For lentivirus packaging, the corresponding plasmids, along with packaging plasmids psPAX2 and PMD2.G (Addgene), were co-transfected into 293 T cells using the Lipofectamine 2000 Transfection Kit (11668019, Invitrogen, USA). Virus particles were collected 48 h after transfection. Cells were infected with the virus by using polybrene (TR-1003, Sigma, USA) and selected with puromycin (HY-B1743A, MCE, USA).

### Cell cycle assay

Cell cycle distribution was analyzed by flow cytometry using a Cell Cycle Detection Kit (Wanlei Biotechnology, Shenyang, China).

### Animal experiments

Male NOD-SCID mice (5–8 weeks) were purchased from Guangdong GemPharmatech Co., Ltd. with quality certification (Certificate No. 44824700012145 and 44824700029421). They were housed under specific pathogen-free (SPF) conditions at the Experimental Animal Center of Sun Yat-sen University. NOD-SCID mice received intravenous injections of 5 × 10^6^ EGFP/Luc-labeled leukemia cells (MOLM-13 or MOLM-13/AR) in 100 μL PBS. After 10 days, the mice bearing MOLM-13-EGFP/Luc cells were randomized into 3 treatment groups: vehicle, 5 mg/mL W1307, or 15 mg/mL W1307 (i.p., daily). Mice bearing MOLM-13/AR-EGFP/Luc cells were randomized into 4 treatment groups: vehicle, W1307, Ara-C, or a combination of W1307 and Ara-C. Each group contains 6 mice. Sample sizes were determined based on our previous experience and similar well-established studies in the literature. Leukemia progression was monitored in vivo by bioluminescence imaging every 7 days. After two weeks of observation, the study was terminated, and tissues were collected for analysis. The animal experiments in this study adhere to the ARRIVE guidelines.

### Pharmacokinetic study

The pharmacokinetics of W1307 were evaluated in ICR mice (male, *n* = 3) following a single intraperitoneal (i.p.) dose of 10 mg/kg. A dosing formulation was prepared by dissolving W1307 in a vehicle of DMSO, Solutol, and saline. Blood samples (0.05 mL) were collected via the facial vein at 5 min, 15 min, 30 min, 1 h, 2 h, 4 h, 6 h, 8 h, and 24 h post-dose. Plasma concentrations of W1307 were determined by LC-MS/MS. In brief, 10 µL of plasma was protein-precipitated with 200 µL of an internal standard solution in methanol:acetonitrile (1:1, v/v), followed by vortexing, centrifugation, and dilution. The supernatant was analyzed by LC-MS/MS. Key pharmacokinetic parameters were calculated from the plasma concentration-time data using non-compartmental analysis in WinNonlin software.

### Statistical analysis

All statistical analyses were performed using GraphPad Prism 8.0 software (GraphPad Software Inc., La Jolla, CA, USA). Quantitative data are presented as the mean ± standard deviation (SD) from at least three independent biological replicates unless otherwise stated. Statistical differences between the two groups were evaluated using an unpaired, two-tailed Student’s t-test. Pearson’s correlation analysis was utilized to assess the correlations between genes. Survival analysis was performed using the Kaplan-Meier method, with group comparisons evaluated via the log-rank test. For heatmap visualizations, data were Z-score normalized and represented by color intensity. The exact sample sizes (n) and specific statistical significance thresholds (e.g., *p < 0.05, **p < 0.01, ***p < 0.001) are explicitly detailed in the respective figure legends. A *p*-value of < 0.05 was considered statistically significant.

## Results

### Active lipid metabolism is associated with chemotherapy resistance of leukemia cells

**Fig. 1:**
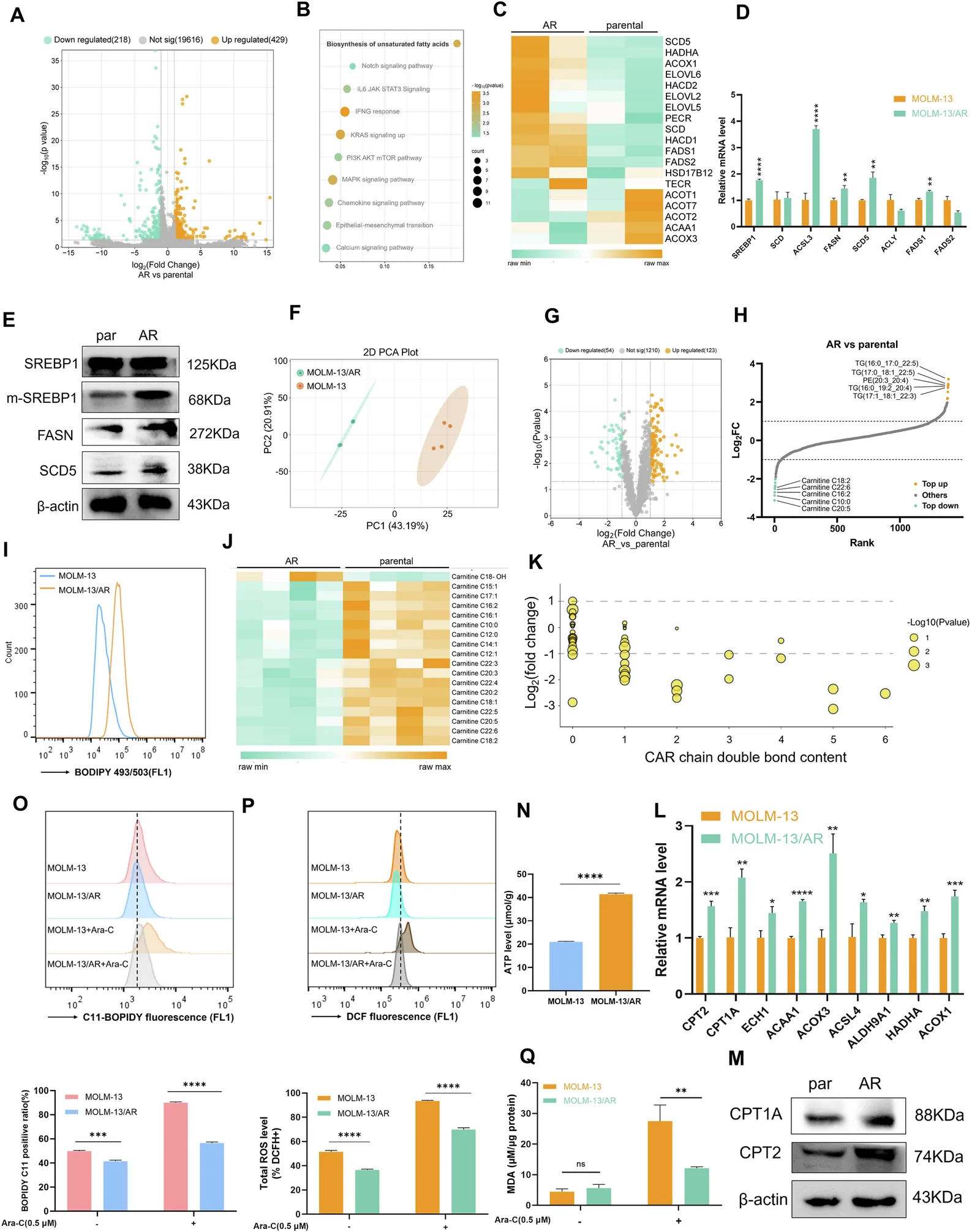
Active lipid metabolism is associated with chemotherapy resistance of leukemia cells. **A** Volcano plot of differentially expressed genes (DEGs) between MOLM-13/AR and MOLM-13 cells (*p* < 0.05; | log2 (Fold Change) | > 2). **B** Pathway enrichment analysis of genes upregulated in MOLM-13/AR cells displayed in a bubble chart. **C** Heatmap showing gene expression profiles in fatty acid biosynthesis pathway. **D** RT-PCR analysis of genes associated with lipid biosynthesis in MOLM-13/AR and MOLM-13 cells. **E** Immunoblot analysis of the indicated proteins in MOLM-13/AR and MOLM-13 cells. **F** Principal component analysis (PCA) of lipidomic data in MOLM-13/AR and MOLM-13 cells. **G** Volcano plot comparing lipid differences between the two groups. **H** Cumulative distribution of differential lipid metabolites between analysis groups. **I** BODIPY 493/503 staining to assess neutral lipid content in MOLM-13 and MOLM-13/AR cells. **J** Heatmap showing differential acylcarnitine levels between analysis groups. **K** Changes in the chain double bond content of acylcarnitines between the analysis groups. **L** RT-PCR analysis of genes involved in fatty acid β-oxidation in MOLM-13/AR and MOLM-13 cells. **M** Immunoblot analysis of the indicated proteins in MOLM-13/AR and MOLM-13 cells. **N** Intracellular ATP levels in MOLM-13 and MOLM-13/AR cells. **O** Flow cytometry analysis of lipid ROS in MOLM-13 and MOLM-13/AR cells, with or without Ara-C treatment for 48 h. Quantification of C11-postive cells is shown. **P** Flow cytometry analysis of ROS in MOLM-13 and MOLM-13/AR cells, with or without Ara-C treatment for 48 h. Quantification of total ROS levels is shown. **Q** Intracellular MDA levels in MOLM-13 and MOLM-13/AR cells, with or without Ara-C treatment for 48 h. *P < 0.05; **P < 0.01; ***P < 0.001; ****P < 0.0001; two-tailed t test. Data are presented as mean ± SD and are representative of three independent experiments.

To clarify the chemoresistance mechanisms in leukemia, we generated an Ara-C-resistant MOLM-13 variant (MOLM-13/AR) by continuous exposure to low concentrations of Ara-C, with a resistance index of 19.84 (Fig. S1A). Total RNA sequencing (RNA-seq) revealed 429 genes increased and 218 decreased in MOLM-13/AR compared to parental cells (Fig. 1A). Gene enrichment analysis identified alterations in pathways, including the biosynthesis of unsaturated fatty acids and the IL6-JAK-STAT3 signaling pathway (Fig. 1B). Specifically, key metabolic enzyme genes participating in fatty acid biosynthesis were markedly up-regulated (Fig. 1C). To identify the upstream driver of this lipid metabolic shift, we performed Gene Set Enrichment Analysis (GSEA), which indicated the global hyperactivation of the SREBP pathway in MOLM-13/AR cells (Fig. S1B). As master transcription factors, SREBPs, encoded by the SREBF1 and SREBF2 genes, modulate fatty acid and cholesterol biosynthesis. Indeed, our transcriptomic data revealed a significant increase in the TPM values of SREBF1 (Fig. S1C). Experimental validation confirmed the upregulation of SREBP1 and its downstream lipid metabolism-related enzymes in MOLM-13/AR cells (Fig. 1D, E, S1E). Furthermore, Kaplan–Meier survival analysis revealed that higher expression of SREBF1 and its downstream gene SCD5 correlated with worse AML prognosis (Fig. S1D), prompting us to prioritize SREBP1 for further functional validation. To assess lipid metabolism alterations, we conducted lipidomics analysis using LC–MS/MS on MOLM-13 and MOLM-13/AR cells. PCA and OPLS-DA results revealed significant lipid changes (Fig. 1F and S1F). Lipidomics identified 177 differentially regulated lipids, with 123 upregulated and 54 downregulated in MOLM-13/AR cells (Fig. 1G). Lipidomics profiling revealed an enrichment of key membrane components, including phosphatidylcholine (PC), phosphatidylethanolamine (PE), phosphatidylglycerol (PG), phosphatidylserine (PS) in MOLM-13/AR cells (Fig. S1I), alongside an increased double bond content in PC chains (Fig. S1H). Furthermore, this alteration of membrane lipid composition was accompanied by increased cholesterol levels, as demonstrated by biochemical assays (Fig. S1G). The elevation of these critical membrane components suggests alterations in membrane fluidity. These changes may contribute to chemotherapy resistance by altering membrane permeability and impacting the function of drug transporters, thereby restricting the intracellular influx of Ara-C [28]. The distribution plot and the heatmap indicated increased triglyceride (TG) levels in MOLM-13/AR cells (Fig. 1H). Consistently, BODIPY 493/503 staining further confirmed a markedly elevated intracellular neutral lipid content (Fig. 1I).

Interestingly, the five most downregulated lipids in MOLM-13/AR cells were all acylcarnitines (Fig. 1H), which are critical intermediates in fatty acid β-oxidation (FAO). Lipidomics analysis showed that acylcarnitines and their chain double bond content were significantly reduced (Fig. 1J, K), indicating altered FAO levels. RT-PCR of FAO-related metabolic enzymes confirmed that transcript levels of these genes were higher in MOLM-13/AR compared to parental cells (Fig. 1L), and protein levels of carnitine palmitoyltransferase (CPT) were elevated (Fig. 1M). Furthermore, ATP production was remarkably increased in MOLM-13/AR (Fig. 1N). Lipid metabolism and SREBP1 signaling pathway regulate cellular redox homeostasis [29, 30]. We further quantified total and lipid-ROS, and found lower levels of both types of ROS in MOLM-13/AR cells compared to parental cells. ROS levels only slightly increased after chemotherapy treatment (Fig. 1O, P). Levels of Malondialdehyde (MDA), a reliable marker of lipid peroxidation, were similar in both cell types at rest, but Ara-C treatment resulted in lower MDA levels in MOLM-13/AR cells than in parental cells (Fig. 1Q), indicating that the resistant cells possess an enhanced capacity to mitigate chemotherapy-induced lipid peroxidation. To investigate how de novo lipogenesis promotes chemo-resistance, we knocked down SREBP1 in MOLM-13/AR cell lines using shRNA (shSREBP1 #1 and #2) (Fig. S1J, K). Notably, neutral lipid content and the IC50 for Ara-C were significantly reduced in SREBP1-knockdown cells (Fig. S1L, M). In contrast, forced expression of SREBP1 in parental MOLM-13 cell lines increased the neutral lipid content and decreased sensitivity to Ara-C (Fig. S1O-Q). Given that lipid catabolism was also robustly elevated, we next evaluated the role of CPT2, a rate-limiting enzyme essential for fatty acid oxidation, in resistant AML cells. Knockdown of CPT2 distinctly altered the intracellular neutral lipid content and partially sensitized cells to Ara-C (Fig. S1R-T), whereas overexpression of CPT2 conferred resistance (Fig. S1U-W).

These results indicate that both de novo lipogenesis and fatty acid oxidation collaboratively maintain an active lipid metabolic homeostasis to support chemoresistance. Therefore, targeting lipid metabolism represents a promising therapeutic strategy to overcome chemoresistance in AML.

### Inhibition of STAT3 disrupts lipid homeostasis and triggers toxic lipid overload in chemoresistant AML cells

**Fig. 2:**
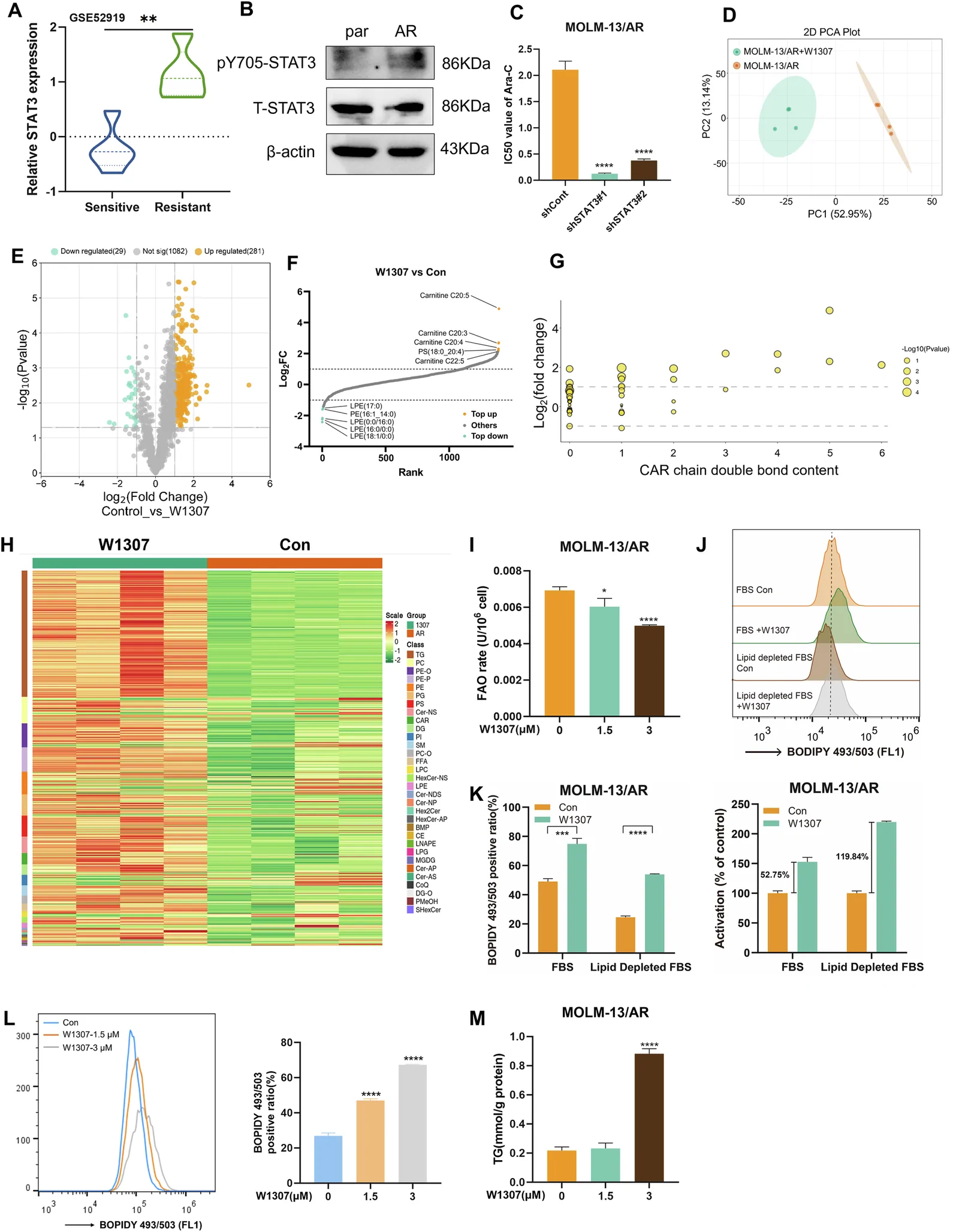
Inhibition of STAT3 disrupts lipid homeostasis and triggers toxic lipid overload in chemoresistant AML cells. **A** Relative STAT3 expression levels in Ara-C-sensitive and Ara-C-resistant patient samples from GSE52919. **B** Immunoblot analysis of STAT3 phosphorylation (pY705) in MOLM-13 and MOLM-13/AR cells. **C** Half-maximal inhibitory concentration (IC50) of Ara-C determined by CCK8 assay in MOLM-13/AR and STAT3-knockdown MOLM-13/AR cells. **D** PCA of lipidomic analysis comparing MOLM-13/AR and MOLM-13/AR + W1307 treatment groups. **E** Volcano plot showing lipid differences between the analysis groups. **F** Cumulative distribution of differential lipid metabolites between the groups. **G** Changes in the chain double bond content of acylcarnitines between the groups. **H** Heatmap displaying the differential lipid contents. **I** FAO rates were measured in MOLM-13/AR cells treated with 0, 1.5, or 3 μM W1307 for 48 h. **J** BODIPY 493/503 staining of W1307-induced intracellular neutral lipid accumulation in MOLM-13/AR cells cultured in standard FBS or lipid-depleted FBS. **K** Quantification of the percentage of BODIPY-positive cells (Left). Fold-change in normalized BODIPY activation relative to the respective untreated control in each serum condition (Right). **L** BODIPY 493/503 staining for neutral lipid content in MOLM-13/AR cells treated with 0, 1.5, or 3 μM W1307 for 48 h. **M** Intracellular TG levels in MOLM-13/AR cells treated with 0, 1.5, or 3 μM W1307 for 48 h. *P < 0.05; **P < 0.01; ***P < 0.001; ****P < 0.0001; two-tailed t test. Data are presented as mean ± SD and are representative of three independent experiments.

While targeting individual metabolic nodes, such as SREBP1 or CPT2, can partially reverse Ara-C resistance, the intrinsic complexity and compensation of lipid metabolic networks limit the long-term efficacy of single target interventions [31]. We therefore sought to identify an upstream master regulator capable of broadly governing this global metabolic rewiring. As shown in Fig. 1, MOLM-13/AR cells exhibited upregulated IL6-JAK-STAT3 pathway. To further identify the clinical relevance of STAT3 in chemotherapy-resistant AML, we reanalyzed GSE52919 transcriptomic data and identified that STAT3 upregulation in Ara-C-resistant samples compared to sensitive samples (Fig. 2A). Consistently, Western blotting and GSEA analysis confirmed the upregulation of pY705-STAT3 and IL6-JAK-STAT3 pathway in MOLM-13/AR cells (Fig. 2B, S2A). To evaluate the functional role of STAT3 in resistance, we generated stable STAT3-knockdown MOLM-13/AR cell lines using shRNA (shSTAT3 #1 and #2) (Fig. S2B-C). STAT3 knockdown significantly enhanced sensitivity to Ara-C (Fig. 2C), exhibiting a more profound sensitizing effect than the depletion of either SREBP1 or CPT2 alone.

To define the global lipidomic alterations associated with STAT3 inhibition, we compared lipidomics data from MOLM-13/AR cells with and without treatment of W1307, a specific STAT3 inhibitor (Figs. 2D, S2D). This analysis identified 310 differential lipids, with the vast majority (281) being significantly upregulated after W1307 treatment (Fig. 2E, H). Notably, four of the top five upregulated lipids in the W1307-treated group were acylcarnitines (Fig. 2F), which are indicative of impaired fatty acid β-oxidation and mitochondrial dysfunction [30]. Unlike the active lipid metabolic homeostasis observed in untreated resistant cells, this massive accumulation of metabolic intermediates represents a toxic metabolic dysregulation. Specifically, polyunsaturated acylcarnitines were markedly enriched following STAT3 inhibition (Figs. 2G, S2E). To directly confirm the blockade of lipid catabolism, we measured the FAO rate and found that W1307 significantly suppressed lipid oxidation (Fig. 2I). This severe catabolic arrest transforms the intracellular lipid pool from a pro-survival energy reservoir into a source of lipotoxicity. Furthermore, we found that W1307-induced neutral lipid accumulation persisted in cells cultured in lipid-depleted FBS, effectively excluding the possibility that this lipid overload was driven by increased exogenous lipid uptake (Fig. 2J, K). Consistently, neutral lipid content and TG levels increased in STAT3-knockdown or W1307-treated MOLM-13/AR cells (Fig. 2L, M, S2F). Together, these data highlight STAT3 as a key regulator of AML chemotherapy resistance, and suggest that targeting STAT3 disrupts overall lipid homeostasis, leading to net intracellular lipid accumulation in chemotherapy-resistant cells.

### Discovery of a novel and selective STAT3 inhibitor W1307

**Fig. 3:**
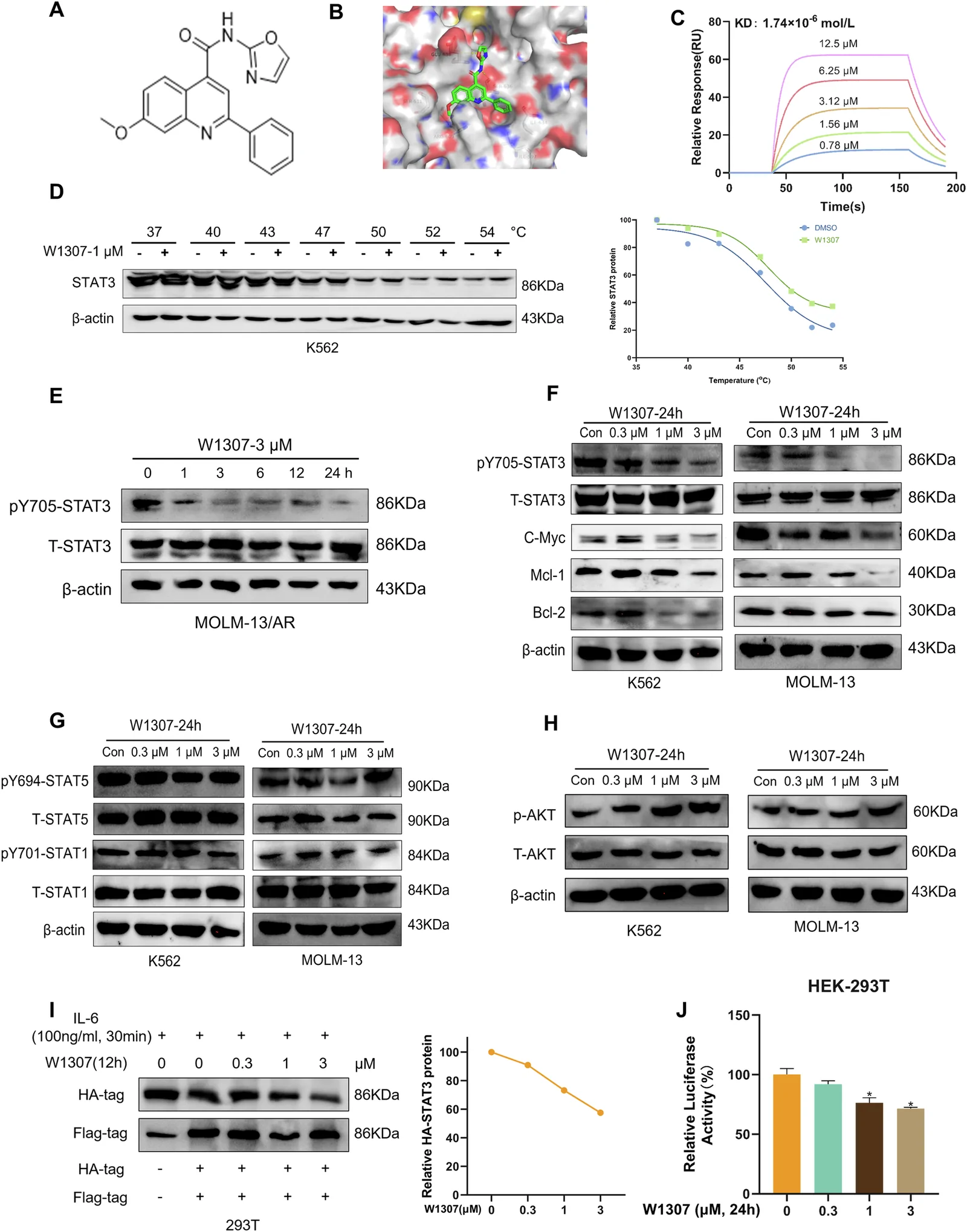
Discovery of a novel and selective STAT3 inhibitor W1307. **A** Chemical structure of W1307. **B** Computational docking model of W1307 bound to the STAT3 protein. The green molecule represents W1307. **C** SPR analysis of the interaction between W1307 and STAT3. **D** Western blot and melting curve analysis of STAT3 protein in CESTA, showing degradation in K562 cells treated with W1307 (1 μM) for 1 h at different temperatures. **E** Immunoblotting analysis of the inhibitory effect of W1307 (3 μM) on STAT3 phosphorylation in MOLM-13/AR cells at the indicated time points. **F** Phosphorylation levels (pY705) of STAT3 and expression of STAT3, c-Myc, Mcl-1, and Bcl-2 in K562 and MOLM-13 cells treated with W1307 at 0, 0.3, 1, and 3 μM for 24 h. **G** Expression levels of pY694-STAT5, STAT5, pY701-STAT1, and STAT1 in K562 and MOLM-13 cells treated with W1307 for 24 h. **H** Protein levels of p-AKT and AKT in K562 and MOLM-13 cells treated with W1307 for 24 h. **I** Immunoprecipitation assay to detect the effect of W1307 on STAT3 dimerization in 293 T cells after 12-hour treatment. Relative HA-STAT3 protein levels are shown. **J** Dual-luciferase reporter assay measuring the effect of W1307 on STAT3 transcriptional activity in 293 T cells after 24-hour treatment. *P < 0.05; two-tailed t test. Data are presented as mean ± SD and are representative of three independent experiments.

Given the established role of STAT3 in AML chemoresistance and lipid metabolism, we developed novel inhibitors targeting this pathway, ultimately selecting W1307 as a potential therapeutic molecule (Fig. 3A) for further investigation. To explore the potential binding mode between W1307 and the STAT3 protein, we conducted docking analysis. The results showed that W1307 binds at the pivotal core for phospho-tyrosine binding (Fig. 3B). To assess the affinity between W1307 and STAT3, we performed SPR assays, which revealed a high binding affinity with a KD value of 1.74 μM (Fig. 3C). Furthermore, we used the CETSA to evaluate drug-protein interactions in native cellular environment. The results demonstrated that W1307 binds to and stabilizes the STAT3 protein in AML cells, confirming a direct interaction (Fig. 3D).

We next investigated the effects of W1307 on the STAT3 signaling pathway. Time-course analysis demonstrated that W1307 inhibited Tyr705 phosphorylation of STAT3 in a time-dependent manner, with significant suppression observed as early as 1 h post-treatment (Fig. 3E). Furthermore, western blot analysis revealed that W1307 dose-dependently inhibited Tyr705 phosphorylation of STAT3 and suppressed the expression of its downstream targets (c-Myc, Mcl-1, and Bcl-2), which regulate cell proliferation, survival, and anti-apoptosis (Fig. 3F), without affecting STAT1/5 phosphorylation (Fig. 3G), nor phospho-AKT levels, the upstream kinase of STAT3 (Fig. 3H). To assess W1307’s effect on STAT3 dimerization, HA- and Flag-tagged STAT3 were co-expressed in 293 T cells for Co-IP analysis. The results showed that W1307 disrupted STAT3-STAT3 dimerization (Fig. 3I). Furthermore, Luciferase reporter assays confirmed that W1307 inhibited STAT3 transcriptional activity (Fig. 3J). To confirm the selectivity of W1307, we generated stable STAT3-knockout AML cell lines using CRISPR/Cas9. W1307’s anti-proliferative effect was attenuated in STAT3-knockout AML cells (Fig. S3A–S3B), indicating that the anti-proliferative effects of W1307 are STAT3-dependent. Collectively, these data suggest that identify W1307 as a selective STAT3-targeting compound with significant inhibitory potency.

### W1307 strongly suppresses AML cell proliferation, survival and induces apoptosis

**Fig. 4:**
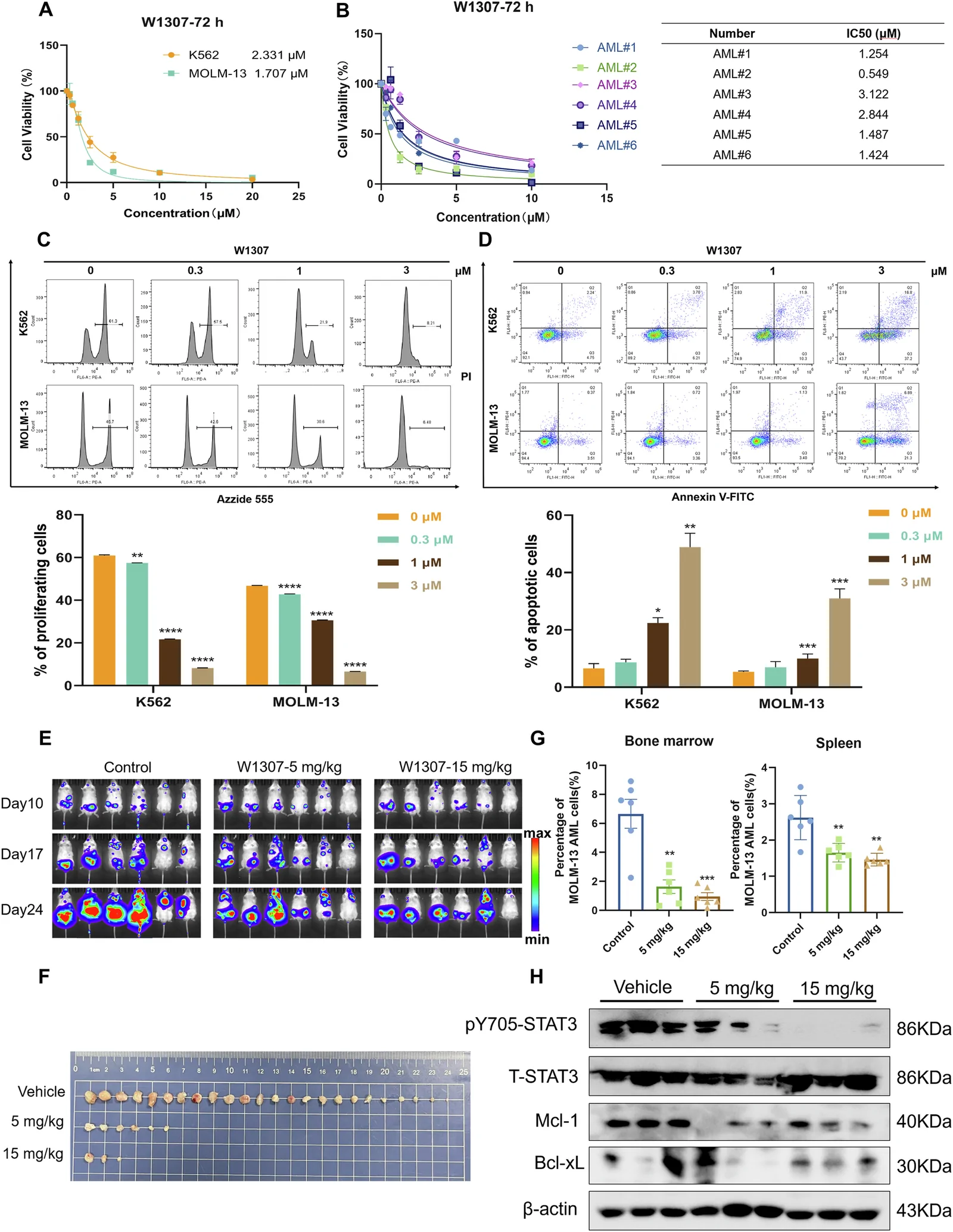
W1307 strongly suppresses AML cell proliferation, survival, and promotes apoptosis. **A** Cell viability measured by CCK8 in K562 and MOLM-13 cells treated with varying concentrations of W1307 for 72 h. **B** Cell viability measured by CCK8 in AML cells from patients treated with varying concentrations of W1307 for 72 h. **C** Flow cytometry analysis of EdU incorporation in K562 and MOLM-13 cells treated with W1307 at varying concentrations for 48 h. Quantification of proliferating cells (%) is shown. **D** Flow cytometry analysis of apoptosis in K562 and MOLM-13 cells treated with W1307 at varying concentrations for 48 h. Quantification of apoptotic cells (%) is shown. **E** Bioluminescence imaging of MOLM-13-EGFP/Luc xenograft mouse models treated with vehicle or W1307 (5 mg/kg and 15 mg/kg) at days 10, 17, and 24 (*n* = 6). **F** Number and representative images of extramedullary tumors harvested from each group at the end of the experiment. **G** Flow cytometry analysis of residual GFP+ cells in bone marrow and spleen (*n* = 6). **H** Immunoblot analysis of indicated proteins in MOLM-13 xenograft tumors treated with vehicle or W1307 (5 mg/kg and 15 mg/kg). *P < 0.05; **P < 0.01; ***P < 0.001; ****P < 0.0001; two-tailed t test. Data are presented as mean ± SD and are representative of three independent experiments.

We next evaluated the anti-leukemia effects of W1307 both in vitro and in vivo. W1307 strongly inhibited the viability of MOLM-13 and K562 cells with hyperactivated STAT3 (Fig. 4A). EdU incorporation assays showed that W1307 effectively suppressed proliferation in MOLM-13 and K562 cells (Fig. 4C). Cell cycle analysis indicated that W1307 caused cell cycle arrest (Fig. S4A). Quantification of apoptosis by Annexin V-FITC/PI staining confirmed W1307’s pro-apoptotic effects in AML cells (Fig. 4D). Additionally, cleavage of caspase-7 and PARP-1, markers of apoptosis, was increased after W1307 treatment (Fig. S4B). To validate clinical potential, W1307 was investigated in primary AML patient cells. At low doses, W1307 potently suppressed viability and triggered apoptosis in primary AML cells (Figs. 4B, S4C). These results demonstrate that W1307 exhibits potent anti-tumor activity in vitro.

Next, we assessed the therapeutic efficacy of W1307 in vivo. We established a murine AML model by intravenously injecting MOLM-13-EGFP/Luc cells into NOD-SCID mice, followed by daily administration of W1307 at doses of 5 or 15 mg/kg. In vivo bioluminescence imaging was performed every 7 days (Fig. S4D). Following 14-day treatment, W1307 exhibited significant, dose-dependent anti-leukemic effects (Fig. 4E). W1307 treatment markedly reduced tumor burden versus vehicle controls (Fig. 4F). Additionally, W1307 treatment reduced the presence of residual MOLM-13-EGFP/Luc cells in the bone marrow and spleen (Figs. 4G, S4E). Histopathological analysis revealed that W1307 treatment reduced the immature blast cells in the blood and a decrease in large basophilic cells in the femoral bone marrow and spleen (Fig. S4F). Furthermore, W1307 treatment suppressed STAT3 signaling (reduced phosphorylation and downstream targets Mcl-1/Bcl-xl) (Fig. 4H) and lipid metabolism proteins (SREBP1, ACSL3, SCD5, CPT2) in tumor tissues (Fig. S4G). These findings suggest that W1307 significantly suppressed aggression of AML in vivo.

### STAT3 globally governs lipid metabolism by transcriptionally regulating both SREBP1 and CPT2

**Fig. 5:**
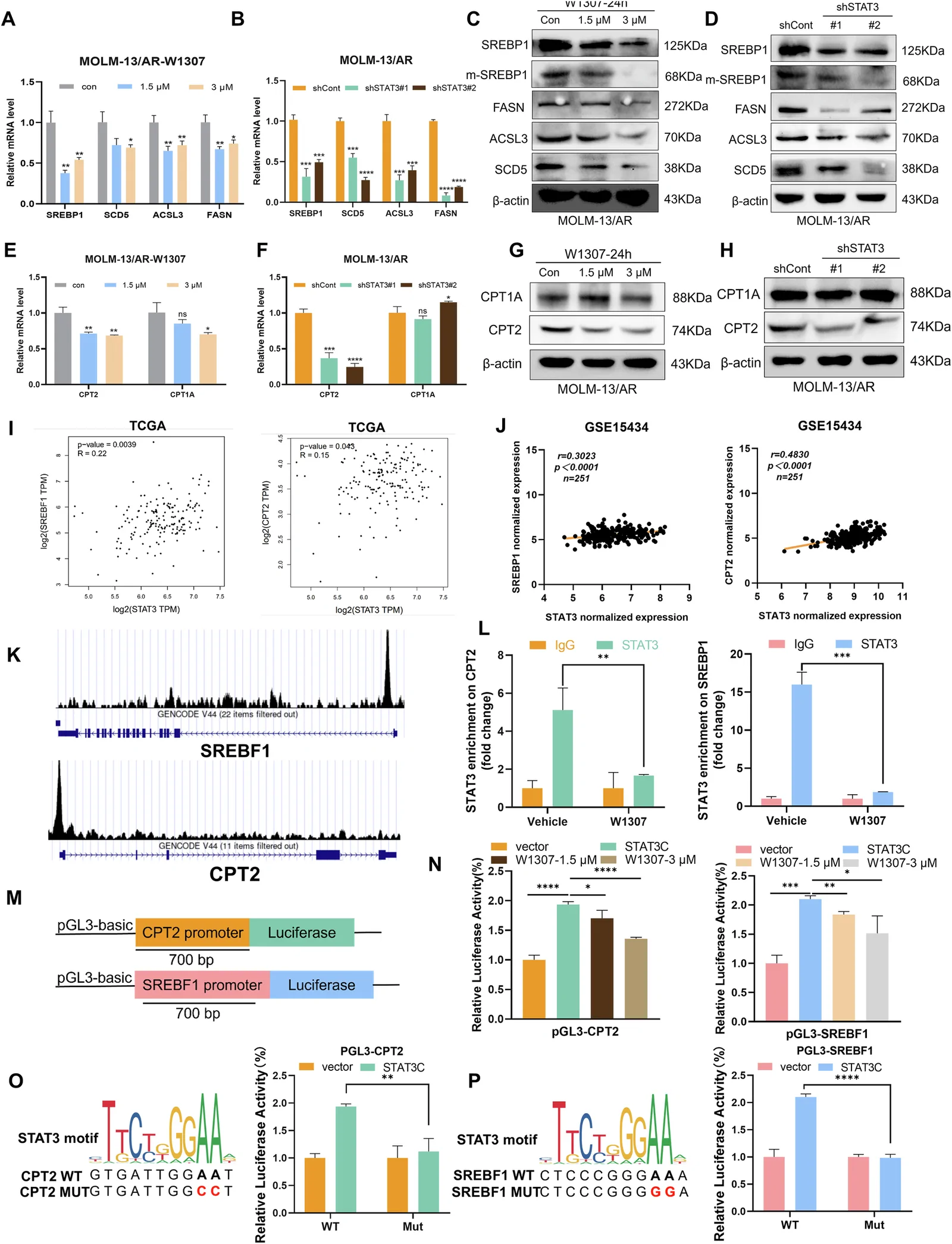
STAT3 mediates lipid metabolism by transcriptionally regulating both SREBP1 and CPT2. **A**, **B** RT-PCR analysis of lipid biosynthesis-related genes in MOLM-13/AR cells treated with W1307 (0, 1.5, and 3 μM) (**A**) and in shCont and shSTAT3 (#1 and #2) MOLM-13/AR cells (**B**). **C**, **D** Immunoblot analysis of relevant proteins in MOLM-13/AR cells treated with W1307 (0, 1.5, and 3 μM) (**C**) and in shCont and shSTAT3 (#1 and #2) MOLM-13/AR cells (**D**). **E**, **F** RT-PCR analysis of CPT2 and CPT1A expression in MOLM-13/AR and MOLM-13 cells, treated with W1307 (0, 1.5, and 3 μM) (**E**), and in shCont and shSTAT3 (#1 and #2) MOLM-13/AR cells (**F**). **G**, **H** Immunoblot analysis of relevant proteins in MOLM-13/AR cells treated with W1307 (0, 1.5, and 3 μM) (**G**) and in shCont and shSTAT3 (#1 and #2) MOLM-13/AR cells (**H**). **I**, **J** Pearson’s correlation analysis of STAT3 and SREBP1/CPT2 mRNA expression levels in leukemia samples from the TCGA LAML database (**I**) and the GEO database (**J**). **K** Genome browser view of STAT3 binding events at the promoters of SREBP1 and CPT2 genes. **L** ChIP-qPCR analysis of STAT3 enrichment at the promoters of SREBP1 and CPT2 genes in MOLM-13/AR cells treated with vehicle or W1307 for 48 h. **M** Schematic representation of CPT2 and SREBF1 promoter cloning into the luciferase reporter vector. **N** Relative luciferase activity of CPT2 and SREBF1 reporters following STAT3 overexpression or W1307 treatment (1.5 and 3 μM) in HEK-293T cells for 24 h. **O** Sequences of wild-type and mutant CPT2 gene promoters (left); relative luciferase activity in response to STAT3 overexpression (right). **P** Sequences of wild-type and mutant SREBF1 gene promoters (left); relative luciferase activity in response to STAT3 overexpression (right). *P < 0.05; **P < 0.01; ***P < 0.001; ****P < 0.0001; two-tailed t test. Data are presented as mean ± SD and are representative of three independent experiments.

Given that STAT3 blockade severely disrupted overall lipid homeostasis (Fig. 2), we hypothesized that STAT3 might simultaneously govern multiple pathways within the lipid metabolic network. To elucidate this, we performed qPCR to detect the effects of STAT3 inhibition on lipid metabolism-related genes. In the context of de novo lipogenesis, we found that SREBP1 expression was dramatically downregulated following both genetic and pharmacological inhibition of STAT3. Several lipid metabolism-related genes regulated by SREBP1, including FASN, ACSL3, and SCD5, were also downregulated (Fig. 5A, B). Consistent with these findings, protein levels of these enzymes were similarly reduced upon STAT3 inhibition (Fig. 5C, D).

Having established its role in lipid biosynthesis, we next investigated how STAT3 impacts fatty acid β-oxidation. Recalling the profound accumulation of acylcarnitines upon STAT3 inhibition (Fig. 2), we examined the carnitine shuttle system. Acylcarnitines are generated from acyl-CoA by CPT1A, which then cross the inner mitochondrial membrane and is subsequently catalyzed by CPT2 to regenerate acyl-CoA [31]. We found that STAT3 inhibition downregulated CPT2 expression but did not significantly affect CPT1A expression (Fig. 5E–H). This differential regulation of the carnitine shuttle enzymes provides a mechanistic basis for the accumulation of acylcarnitines detected in our lipidomics analysis. These results suggest that STAT3 acts as a dual regulator, simultaneously affecting lipid biosynthesis via SREBP1 and lipid catabolism via CPT2 in Ara-C-resistant AML cells.

To investigate how STAT3 regulates SREBP1 and CPT2 expression, we performed multi-database analysis (TCGA/GEO) revealing their positive co-expression (Fig. 5I, J). ChIP-seq data showed distinct binding peaks in the promoters of SREBF1 and CPT2 (Fig. 5K). ChIP-qPCR assays confirmed that STAT3 directly binds to the promoters of these genes, and treatment with W1307 inhibited this binding (Fig. 5L). Luciferase reporter assays with cloned CPT2/SREBF1 promoters confirmed the transcriptional regulation of STAT3 (Fig. 5M, N). Mutation of STAT3-binding motifs attenuated CPT2/SREBF1 promoter activation (Fig. 5O, P). Together, these data demonstrate that STAT3 transcriptionally regulates SREBP1 and CPT2 expression in MOLM-13/AR cells.

### Inhibition of STAT3 induces lipotoxicity via SREBP1/CPT2 axis-mediated lipid metabolic dysregulation

**Fig. 6:**
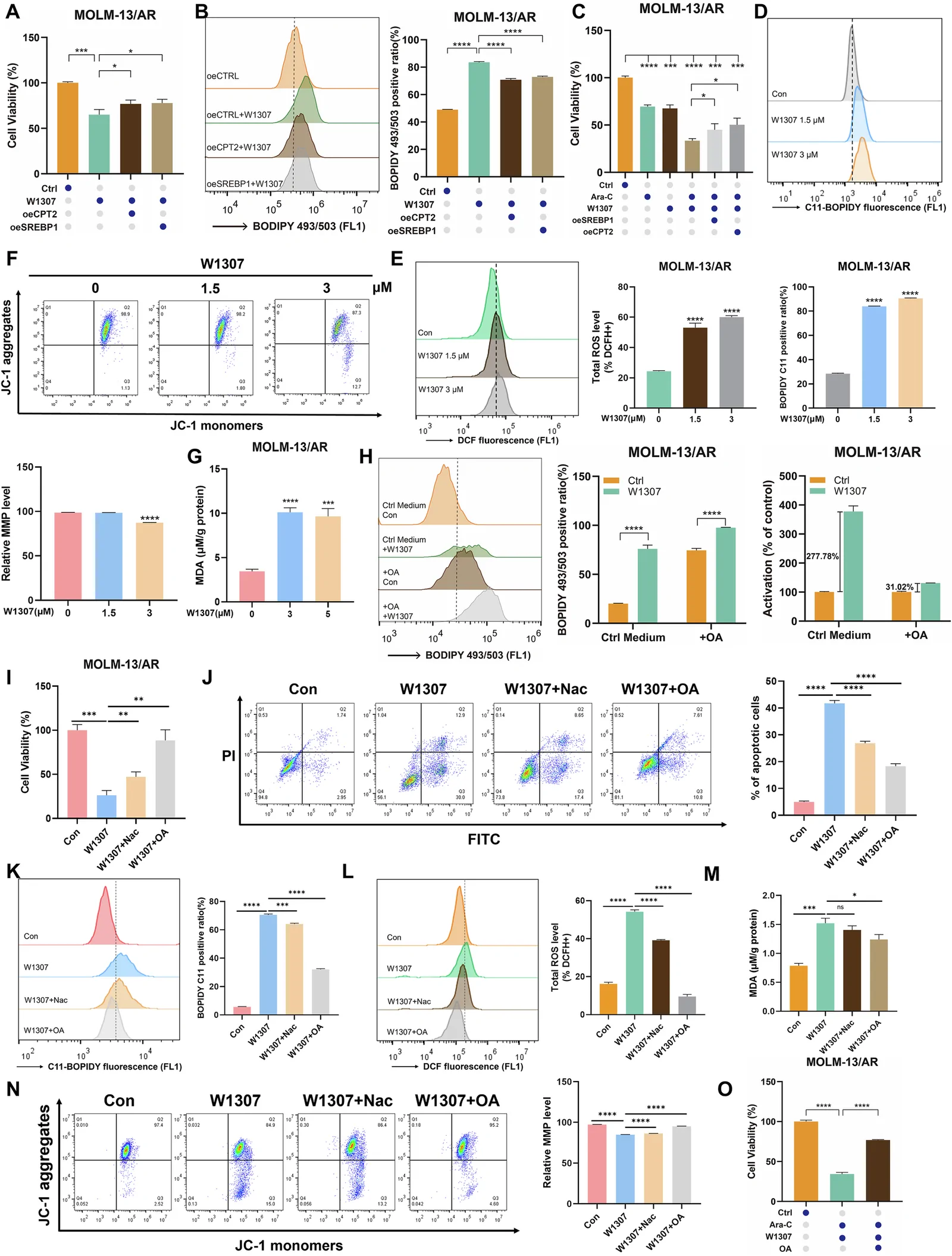
Inhibition of STAT3 induces lipotoxicity via SREBP1/CPT2 axis-mediated lipid metabolic dysregulation. **A** Cell viability of MOLM-13/AR cells treated with W1307, with or without the overexpression of CPT2 or SREBP1. **B** Representative flow cytometry histograms (left) and corresponding quantitative analysis (right) of intracellular neutral lipids assessed by BODIPY 493/503 staining. **C** The cell viability of MOLM-13/AR cells treated with Ara-C, W1307, or their combination, with or without the overexpression of SREBP1 or CPT2. **D** Flow cytometry analysis of lipid ROS in MOLM-13/AR cells treated with W1307 (0, 1.5, and 3 μM) for 48 h (Upper). Quantification of the C11-positive cell ratio (%) is shown (Lower). **E** Flow cytometry analysis of ROS levels in MOLM-13/AR cells treated with W1307 (0, 1.5, and 3 μM) for 48 h (Left). Quantification of total ROS levels is shown (Right). **F** Mitochondrial membrane potential (MMP) analysis in MOLM-13/AR cells treated with W1307 (0, 1.5, and 3 μM) for 48 h using JC-1 probe. **G** Intracellular MDA levels in MOLM-13/AR cells treated with W1307 (0, 3, and 5 μM) for 48 h. **H** BODIPY 493/503 staining of W1307-induced intracellular neutral lipid accumulation in MOLM-13/AR cells in the presence or absence of OA (50 μM) (Left). Quantification of the percentage of BODIPY-positive cells (Middle). Fold-change in normalized BODIPY activation relative to the respective untreated control in each serum condition (Right). **I** Cell viability measured by CCK8 in MOLM-13/AR cells treated with or without W1307 (3 μM) in the presence or absence of NAC (2 mM) or OA (50 μM) for 48 h. **J** Flow cytometry analysis of apoptosis in MOLM-13/AR cells treated with or without W1307 (3 μM) in the presence or absence of NAC (2 mM) or OA (50 μM) for 48 h. Quantification of apoptotic cells (%) is shown. **K** Flow cytometry analysis of lipid ROS in MOLM-13/AR cells treated with or without W1307 (3 μM) in the presence or absence of NAC (2 mM) or OA (50 μM) for 48 h. Quantification of C11-positive cells is shown. **L** Flow cytometry analysis of ROS in MOLM-13/AR cells treated with or without W1307 (3 μM) in the presence or absence of NAC (2 mM) or OA (50 μM) for 48 h. Quantification of total ROS levels is shown. **M** MDA levels in MOLM-13/AR cells treated with or without W1307 (3 μM) in the presence or absence of NAC (2 mM) or OA (50 μM) for 48 h. **N** Mitochondrial membrane potential analysis in MOLM-13/AR cells treated with or without W1307 (3 μM) in the presence or absence of NAC (2 mM) or OA (50 μM) for 48 h using JC-1 probe. **O** The cell viability of MOLM-13/AR cells treated with the combination of Ara-C and W1307, with or without OA (50 μM). *P < 0.05; **P < 0.01; ***P < 0.001; ****P < 0.0001; two-tailed t test. Data are presented as mean ± SD and are representative of three independent experiments.

To confirm whether W1307 exerts its anti-leukemic effects through the STAT3-SREBP1/CPT2 axis, we performed genetic rescue assays. Exogenous overexpression of either SREBP1 or CPT2 in W1307-treated MOLM-13/AR cells effectively reversed cell viability and prevented the intracellular lipid overload induced by STAT3 inhibition (Fig. 6A, B). Moreover, restoring these metabolic regulators reversed the sensitizing effect of W1307 to Ara-C (Fig. 6C), confirming that W1307 acts primarily by targeting STAT3-SREBP1/CPT2 signaling.

Having established this molecular dependency, we next explored the downstream cellular consequences of this targeted metabolic disruption. Previous studies found that STAT3 inhibition results in lipid accumulation within cells, which can induce lipotoxicity. Lipotoxicity typically manifests as free fatty acids accumulation, leading to increased ROS and subsequent oxidative damage to cellular components, including mitochondrial dysfunction and endoplasmic reticulum (ER) stress [32]. To determine if W1307 blockade induces such stress, we exposed cells to W1307 and observed a significant increase in both total and lipid ROS (Fig. 6D, E). It was reported that elevated ROS production leads to mitochondrial dysfunction [33, 34]. We also observed a reduction in mitochondrial membrane potential (MMP) in MOLM-13/AR cells following W1307 treatment (Fig. 6F). Additionally, STAT3 inhibition upregulated malondialdehyde (MDA) levels, a marker of lipid peroxidation (Fig. 6G), confirming the onset of lipotoxic stress.

To validate that this STAT3 inhibition-induced lipotoxicity is the direct executor of cell death, we conducted pharmacological rescue experiments. We utilized oleic acid (OA), a monounsaturated fatty acid known to mitigate oxidative stress, and N-acetyl-L-cysteine (NAC), a well-established antioxidant [35]. Notably, BODIPY 493/503 staining revealed that exogenous OA supplementation successfully attenuated the W1307-induced intracellular neutral lipid accumulation (Fig. 6H). Consequently, both NAC and OA effectively prevented cell death induced by W1307 (Fig. 6I, J). Furthermore, the combination of NAC or OA with W1307 reversed the effects of STAT3 inhibition on ROS formation, MDA accumulation, and MMP reduction (Fig. 6K–N). Most importantly, the addition of OA also reversed the sensitizing effect of W1307, restoring Ara-C resistance in these cells (Fig. 6O).

Collectively, these genetic and pharmacological evidence establish a mechanistic axis: W1307 inhibits STAT3, leading to the differential downregulation of SREBP1 and CPT2. This disruption of metabolic homeostasis induces cytotoxic lipid overload and triggers lipotoxic cell death, ultimately serving as an effective strategy to overcome chemoresistance in AML.

### Synergistic efficacy of W1307 with Ara-C and its clinical validation in chemoresistant AML

**Fig. 7:**
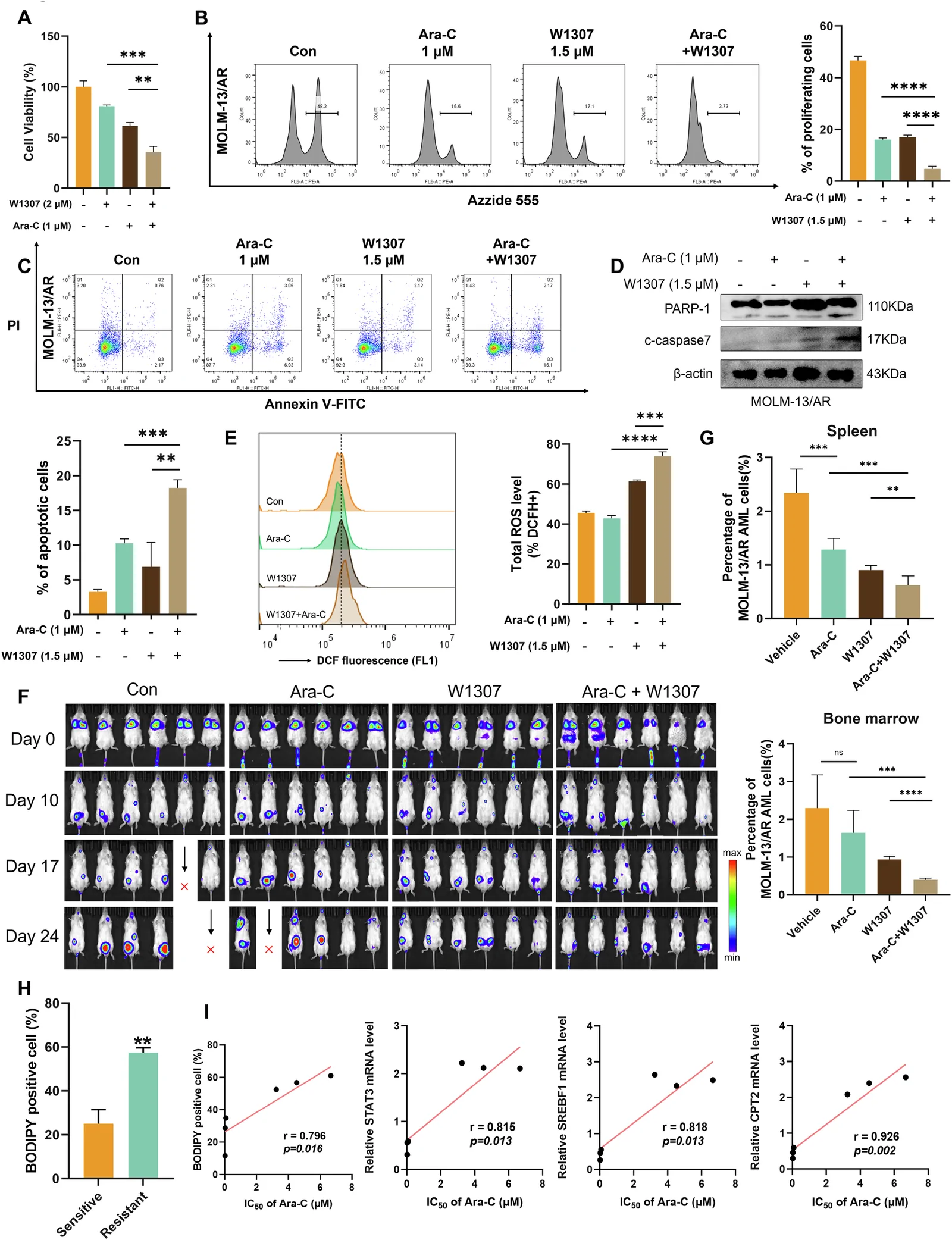
Synergistic efficacy of W1307 with Ara-C and its clinical validation in chemoresistant AML. **A** Cell viability measured by CCK8 in MOLM-13/AR cells treated with Ara-C (1 μM) and/or W1307 (1.5 μM) for 72 h. **B** Flow cytometry analysis of EdU incorporation in MOLM-13/AR cells treated with Ara-C (1 μM) and/or W1307 (1.5 μM) for 48 h. Quantification of proliferating cells (%) is shown. **C** Flow cytometry analysis of apoptosis in MOLM-13/AR cells treated with Ara-C (1 μM) and/or W1307 (1.5 μM) for 48 h. Quantification of apoptotic cells (%) is shown. **D** Expression levels of apoptosis-related proteins cleaved Caspase-7 and PARP-1 in MOLM-13/AR cells treated with Ara-C (1 μM) and/or W1307 (1.5 μM) for 48 h. **E** Flow cytometry analysis of ROS in MOLM-13/AR cells treated with Ara-C (1 μM) and/or W1307 (1.5 μM) for 48 h. Quantification of total ROS levels is shown. **F** Bioluminescence imaging of MOLM-13/AR-EGFP/Luc xenograft models treated with vehicle, Ara-C (30 mg/kg), W1307 (5 mg/kg), or their combination, as indicated, at days 0, 10, 17, and 24. (*n* = 6). **G** Flow cytometry analysis of residual GFP+ cells in the bone marrow and spleen. (*n* = 6). **H** The intracellular neutral lipid content of sensitive and resistant samples. **I** Correlation analysis between the IC50 values of Ara-C and the BODIPY positive cell ratio, as well as the relative mRNA levels of STAT3, SREBF1, and CPT2. *P < 0.05; **P < 0.01; ***P < 0.001; ****P < 0.0001; two-tailed t test. Data are presented as mean ± SD and are representative of three independent experiments.

Having delineated the mechanism by which W1307 induces lipotoxic cell death via the STAT3-SREBP1/CPT2 axis in vitro, we next sought to explore its therapeutic translation. We hypothesized that targeting this specific metabolic vulnerability with W1307 could offer an innovative synergistic strategy for chemotherapy-resistant AML. Indeed, we observed that the W1307-Ara-C combination demonstrated significantly greater anti-proliferative effects than individual drugs (Fig. 7A, B). To quantitatively evaluate this interaction, we applied the Highest Single Agent (HSA) reference model, which confirmed a strong synergistic effect between W1307 and Ara-C, as evidenced by an HSA synergy score of 13.083 (Fig. S5A). Moreover, W1307 enhanced the apoptosis-inducing effect of Ara-C in MOLM-13/AR cells, consistently with the upregulated apoptotic proteins (Fig. 7C, D). Additionally, the combination therapy induced higher ROS accumulation than monotherapy (Fig. 7E).

A similar synergistic effect was observed in a MOLM-13/AR-EGFP/Luc-derived chemo-resistant AML model (Figs. 7F, S5B, C). The combined treatment group exhibited superior anti-leukemic activity, effectively reducing the number of residual MOLM-13/AR-EGFP/Luc cells in the bone marrow and spleen and decreasing spleen weight (Figs. 7G, S5D). Histopathological analysis confirmed these findings (Fig. S5E). Oil Red O staining of liver and spleen tissue showed no significant impact on lipid metabolism in these organs, suggesting that the combination treatment does not adversely affect non-target tissues (Fig. S5F).

To provide a pharmacological rationale for the robust in vivo efficacy observed at this dosage, we conducted a preliminary pharmacokinetic (PK) profiling of W1307. Following a single intraperitoneal administration of 10 mg/kg in mice, W1307 exhibited rapid absorption (Tmax = 0.25 h) and favorable systemic exposure, reaching a maximum plasma concentration (Cmax) of 4300 ng/mL (Table S5). Crucially, this translates to a concentration of approximately 12.46 µM, which is sufficient to achieve the in vitro therapeutic threshold required to suppress STAT3. These PK data validate that an adequate, therapeutically relevant concentration of the drug reaches the tumor microenvironment to drive the observed in vivo antitumor effects.

Finally, to validate the clinical relevance of our mechanistic findings, we evaluated primary samples derived from AML patients. Based on their ex vivo Ara-C IC50 values, patient samples were stratified into Ara-C-sensitive and Ara-C-resistant cohorts (Fig. S5G). Notably, the Ara-C-resistant primary samples exhibited higher intracellular neutral lipid content compared to the sensitive counterparts (Figs. 7H, S5H). Furthermore, correlation analyses revealed that the degree of Ara-C resistance was positively correlated not only with neutral lipid levels but also with the mRNA expression of STAT3, SREBF1, and CPT2 (Figs. 7H, S5I). These data from primary patient samples corroborate our in vitro results, providing preliminary evidence that the STAT3-SREBP1/CPT2 lipid metabolic axis is clinically critical for AML chemoresistance.

Overall, these data demonstrate that combining W1307 with Ara-C may offer a promising strategy for treating chemotherapy-resistant AML.

## Discussion

AML is a prevalent adult leukemia, and understanding its molecular pathogenesis is critical for developing effective treatments. While Ara-C remains frontline AML therapy, acquired resistance often results in clinical relapse [1, 36]. Resistance to Ara-C is associated with various mechanisms, including altered drug absorption and metabolism, overexpression of anti-apoptotic proteins, enhanced DNA repair, the presence of leukemic stem cells (LSCs), and metabolic reprogramming [3, 37, 38]. Growing evidence implicates that alterations in specific metabolites and metabolic pathways play a crucial role in leukemogenesis and chemoresistance. Specifically, dysregulated lipid metabolism has been identified as a driver of survival and drug resistance across various malignancies, prompting increasing interest in targeting lipid metabolism as a therapeutic approach [12, 26, 39]. However, current evidence suggests that targeting individual metabolic pathways or single enzymes yields limited efficacy due to the intrinsic compensation of cellular metabolism [31].

To sustain rapid proliferation and survival, malignant cells exhibit profound metabolic adaptations, particularly in lipid networks governing structural and energetic demands. This metabolic rewiring fuels tumor progression, metastasis, and therapy resistance. In this study, we found that lipid metabolism is activated in AML chemo-resistant cells. This is characterized by the upregulation of SREBP1-mediated lipogenesis and CPT2-mediated fatty acid oxidation, leading to increased intracellular lipid levels. This adaptive lipid increase created a pro-survival environment. Accordingly, resistant cells exhibited significantly lower levels of total ROS, lipid ROS, and MDA, reflecting a capacity to reduce oxidative stress and evade lipid peroxidation. We demonstrate that knocking down either SREBP1 or CPT2 alters intracellular neutral lipid levels and ultimately sensitizes MOLM-13/AR cells to chemotherapy. As expected, overexpressing these factors restored the resistant phenotype, highlighting that therapeutically targeting lipid metabolism represents a promising intervention strategy to overcome chemoresistance in AML. These results align with an emerging consensus that lipid metabolic reprogramming acts as a driver of acquired drug resistance across various malignancies. For instance, enhanced lipid metabolism has been shown to directly promote cisplatin resistance in hepatocellular carcinoma [40], while PPARα-mediated lipid rewiring provides critical metabolic support for anti-EGFR therapy resistance [41]. Furthermore, studies have highlighted that the accumulation of lipid droplet can create a pro-survival environment that shields cancer cells from pharmacological pressure [42, 43]. Consistent with these broad mechanistic insights, our data suggest that the lipid metabolic rewiring and subsequent neutral lipid increase serve as a critical protective mechanism, ultimately conferring Ara-C resistance in AML cells.

Given the limitations of targeting single metabolic nodes, identifying a master regulator capable of globally rewiring this metabolic network is imperative. Beyond its classical role in promoting tumor cell proliferation, STAT3 significantly coordinates metabolic pathways. STAT3 can directly and indirectly regulate bioenergetic pathways, macromolecule biosynthesis, and catabolic processes in cancer cells [44, 45]. Thus, STAT3 represents a potential target for reversing dysregulated metabolism and sensitizing chemotherapy-resistant cells. Indeed, we observed the upregulation of phosphorylated STAT3 (pY705-STAT3) and IL6-JAK-STAT3 pathway in Ara-C-resistant AML cells. Lipidomic analysis revealed significant accumulation of lipids, particularly acylcarnitines, in response to STAT3 inhibition. Mechanistically, our experiments demonstrated that targeting STAT3 directly inhibits the transcription of both SREBP1 and CPT2, thereby concurrently suppressing lipid synthesis and oxidation pathways. Importantly, unlike the pro-survival lipid accumulation driven by active metabolism in untreated resistant cells, W1307 disrupts intracellular lipid metabolic homeostasis, resulting in a severe, global lipid metabolic disorder. Acylcarnitines, known to be lipotoxic, accumulate when mitochondrial β-oxidation is impaired [46, 47]. Thus, acylcarnitine accumulation suggests mitochondrial dysfunction. Nguyen et al. revealed that inhibiting CPT1 to decrease acylcarnitine production rescues MMP, suggesting that high levels of acylcarnitines may cause mitochondrial dysfunction [48]. Consistent with this, we observed mitochondrial dysfunction and increased ROS and MDA production following STAT3 inhibition. Notably, this phenomenon was effectively reversed by exogenous supplementation of oleic acid and NAC, demonstrating that targeting STAT3 directly induces lipotoxicity, which drives oxidative stress and lipid peroxidation. Together, these experiments establish a direct causal pathway: W1307 inhibits STAT3, which differentially downregulates SREBP1 and CPT2, with a net effect favoring accumulation, thereby inducing cytotoxic lipid accumulation, triggering lipotoxic cell death, and ultimately overcoming chemoresistance.

As an oncogenic driver, STAT3 presents a viable therapeutic target for AML [23]. Elevated STAT3 activity is frequently detected in many AML patient samples [49, 50], and predicts poor prognosis [51]. Several STAT3 inhibitors, such as OPB-51602 [52], BBI-608 [53], and C188-9 [23], are investigated in preclinical and early-phase clinical trials. There are also other strategies targeting STAT3, including proteolysis targeting chimeras (PROTACs), antisense oligonucleotide (ASO) inhibitors, small interfering RNAs (siRNAs) [54–56], etc. Despite extensive research, no STAT3 inhibitors are clinically approved for AML, underscoring the demand for novel therapeutics. Here we present W1307, a potent and selective STAT3-targeting compound developed for AML treatment. We demonstrated that W1307 strongly inhibits STAT3 signaling, as evidenced by suppression of downstream target gene expression, inhibition of STAT3 dimerization, and reduced transcriptional activity. Furthermore, W1307 inhibited lipid metabolism and exhibited significant anti-leukemic effects both in vitro and in vivo. We also investigated the therapeutic efficacy of combining W1307 with Ara-C. The W1307 and Ara-C combination synergistically suppressed chemo-resistant AML cell proliferation in vitro and in vivo.

Collectively, we identified key metabolic changes between chemotherapy-resistant and sensitive AML cells and elucidated the critical role of STAT3 in driving chemoresistance. Our findings suggest that targeting STAT3 can overcome chemotherapy resistance by disrupting lipid synthesis and catabolism. The novel STAT3 inhibitor W1307 exhibits potent anti-leukemic efficacy, proposing a therapeutic strategy to overcome chemotherapy resistance in AML. Nevertheless, further investigations are needed to clarify how tumor microenvironmental metabolic alterations modulate cellular treatment responses. Moreover, future studies will incorporate patient-derived xenograft (PDX) models to better account for patient heterogeneity, and will continuously evaluate and optimize the druggability of W1307 for broader clinical translation.

## Supplementary information

Supplementary Materials

Uncropped western blots

## Data Availability

The datasets generated during and/or analyzed during the current study are available from the corresponding author upon reasonable request. RNA-Seq data has been deposited to GEO 327495.
