# Supplementary material for: Targeting STAT3-mediated lipid metabolism reprogramming overcomes chemoresistance in acute myeloid leukemia
Source: Cell Death Dis. 2026 Jun 17;17(1):755. doi: 10.1038/s41419-026-08988-4 (PMC13518950; doi:10.1038/s41419-026-08988-4)
Supplement: Supplementary file 1 [file 41419_2026_8988_MOESM1_ESM.docx]

**Supplementary materials for**

**Targeting STAT3-Mediated Lipid Metabolism Reprogramming Overcomes Chemoresistance in Acute Myeloid Leukemia**

Keren Peng^#1^, Jianshan Mo#^1^, Zhenjiao Yang^#1^, Wen Ding^#1,2^, Jiayu Yan^1^, Shumin Ouyang^1^, Minyuan Lu^1^, Kai zhu^3^, Hongru Yao^4^, Huiqin Chen^5^, Xiongjun Xu^5^, Peibin Yue^6^, Jinjian Lu^7^, Yuanxiang Wang^1^, Shanyi Zhang^4*^, Yandong Wang^2^*, Xiaolei Zhang^1^*

1. National-Local Joint Engineering Laboratory of Druggability and New Drug Evaluation, State Key Laboratory of Anti-Infective Drug Discovery and Development, Guangdong Key Laboratory of Chiral Molecule and Drug Discovery, School of Pharmaceutical Sciences, Sun Yat-sen University, Guangzhou, 510006, China

2. State Key Laboratory of Ophthalmology, Zhongshan Ophthalmic Center, Sun Yat-sen University, Guangzhou 510060, China

3. Innovation Practice Center, Changchun University of Chinese Medicine, Changchun, 130117, China

4. Department of Thoracic Surgery, The Third Affiliated Hospital of Sun Yat-sen University, Guangzhou, 510630, China

5. Department of Medicine, Division of Hematology-Oncology, and Samuel Oschin Comprehensive Cancer Institute, Cedars-Sinai Medical Center, Los Angeles, CA, 90048, USA

6. State Key Laboratory of Quality Research in Chinese Medicine, Institute of Chinese Medical Sciences, University of Macau, Macao, China

#These authors contributed equally to this work.

*To whom correspondence should be addressed:

E-mail: [zhangxlei5@mail.sysu.edu.cn](mailto:zhangxlei5@mail.sysu.edu.cn) (Xiaolei Zhang), [wangyand@mail.sysu.edu.cn](mailto:wangyand@mail.sysu.edu.cn) (Yandong Wang), [zsyscience@163.com](mailto:zsyscience@163.com) (Shanyi Zhang)

**Supplemental FigureS1-4**

**Supplemental TableS1-5**

**Figure S1. Active lipid metabolism is associated with chemotherapy resistance of leukemia cells**

**A**: Cell viability measured by CCK8 in MOLM-13 and MOLM-13/AR cells treated with the indicated concentrations of Ara-C for 72 h. **B**: GSEA analysis of SREBP signaling pathway in MOLM-13/AR cells and MOLM-13 cells. **C**: TPM values of SREBP1 in MOLM-13/AR and MOLM-13 cells determined by RNA-seq. **D**: Kaplan-Meier analysis of AML patients from TCGA project with SREBP1 and SCD5 genes high or low expression levels. **E**: Quantification of lipid biosynthesis-related proteins in parental and Ara-C-resistant AML cells. **F**: Lipidomic analysis was demonstrated by OPLS-DA in MOLM-13/AR cells and MOLM-13 cells. **G**: Intracellular T-CHO level of MOLM-13 cells and MOLM-13/AR cells. **H**: The chain double bond content changes in PC between MOLM-13 cells and MOLM-13/AR cells. **I**: The heatmap of PC, PE, PS, PG contents in MOLM-13 cells and MOLM-13/AR cells. **J-K**: Knockdown of SREBP1 in MOLM-13/AR cells by shRNA. The knockdown effect was verified by western blotting (J) and RT-PCR (K). **L**: BODIPY 493/503 staining for neutral lipid content in MOLM-13/AR cells and SREBP1-knockdown MOLM-13/AR cells. **M**: Half maximal inhibitory concentration of Ara-C determined by CCK8 assay in MOLM-13/AR and SREBP1-knockdown MOLM-13/AR cells. **N-O**: Overexpression of SREBP1 in MOLM-13 cells. The overexpression effect was verified by RT-PCR (N) and western blotting (O). **P**: BODIPY 493/503 staining for neutral lipid content in MOLM-13 cells and SREBP1-overexpression MOLM-13 cells. **Q**: Half maximal inhibitory concentration of Ara-C determined by CCK8 assay in MOLM-13 and SREBP1-overexpression MOLM-13 cells. **R**: Knockdown of CPT2 in MOLM-13/AR cells by siRNA. The knockdown effect was verified by RT-PCR. **S**: BODIPY 493/503 staining for neutral lipid content in MOLM-13/AR cells and CPT2-knockdown MOLM-13/AR cells. **T**: Half maximal inhibitory concentration of Ara-C determined by CCK8 assay in MOLM-13/AR and CPT2-knockdown MOLM-13/AR cells. **U**: Over-expression of CPT2 in MOLM-13 cells by plasmid transfection. The over-expression efficiency was verified by RT-PCR. **V**: BODIPY 493/503 staining for neutral lipid content in MOLM-13 cells and SREBP1-overexpression MOLM-13 cells. **W**: Half maximal inhibitory concentration of Ara-C determined by CCK8 assay in MOLM-13 and SREBP1-overexpression MOLM-13 cells. *P < 0.05; **P < 0.01; ***P < 0.001; ****P < 0.0001; two-tailed t test. Data are presented as mean ± SD and are representative of three independent experiments.

**Figure S2. Inhibition of STAT3 disrupts lipid homeostasis and triggers toxic lipid overload in chemoresistant AML cells**

**A**: GSEA analysis of JAK STAT pathway in MOLM-13/AR cells and MOLM-13 cells. B-C: Knockdown of STAT3 in MOLM-13/AR cells by shRNA. The knockdown effect was verified by western blotting (**B**) and RT-PCR (**C**). **D**: Lipidomic analysis was demonstrated by OPLS-DA in groups: MOLM-13/AR and MOLM-13/AR+W1307. **E**: The heatmap of the differential acylcarnitine levels between analysis groups. **F**: BODIPY 493/503 staining for neutral lipid content in shCtrl and shSTAT3 (#1 and #2) MOLM-13/AR cells. *P < 0.05; **P < 0.01; ***P < 0.001; ****P < 0.0001; two-tailed t test. Data are presented as mean ± SD and are representative of three independent experiments.

**Figure S3. Discovery of a novel and selective STAT3 inhibitor W1307** **A**: The knockout efficiency of STAT3 in MOLM-13 cells verified by western blotting. **B**: Cell viability of MOLM-13 cells and KO-STAT3-MOLM-13 cells with W1307 treatment at different concentrations. *P < 0.05; two-tailed t test. Data are presented as mean ± SD and are representative of three independent experiments.

**Figure S4. W1307 strongly suppresses AML cells proliferation, survival and promotes apoptosis**. **A**: Represent flow cytometry histogram of cell cycle assay in K562 and MOLM-13 cells treated with the indicated concentrations of W1307 for 48 h. Quantification of populations was shown. **B**: The expression changes of apoptosis-related proteins cleavaged-Caspase-7 and PARP-1 in K562 and MOLM-13 cells treated with W1307 at 0, 0.3, 1, 3 μM for 24 h. **C**: Cell apoptosis analyzed by flow cytometry in AML cells derived from patients treated with the indicated concentrations of W1307 for 48 h. Quantification of apoptotic cells (%) was shown. **D**: Illustration of procedure of therapy in AML mouse models. **E**: Illustration of the analysis procedure of residual GFP+ cells of mice (n = 6) by flow cytometry. **F**: Giemsa staining of blood smears and HE staining of spleen and bone marrow. The arrows indicated leukemia-like cells. **G**: Immunoblotting analysis of the indicated proteins in MOLM-13 xenograft tumors after treatment with vehicle or W1307(5 mg/kg and 15 mg/kg). *P < 0.05; **P < 0.01; ***P < 0.001; ****P < 0.0001; two-tailed t test. Data are presented as mean ± SD and are representative of three independent experiments.

**Figure S5. Synergistic efficacy of W1307 with Ara-C and its clinical validation in chemoresistant AML**

**A**: The 2D synergy landscape map illustrates the interaction between W1307 and Ara-C. **B**: Illustration of procedure of therapy in AML mouse models. **C**: Quantitative analysis of total fluorescence counts at experimental end point. **D**: Representative images of spleen harvested from mice of each group at experimental end point. Weights of spleen harvested from mice of each group was shown. **E**: HE staining of spleen and bone marrow. **F**: Oil red O staining of liver and spleen. **G**: Cell viability curves and the calculated IC50 values of primary AML patient samples treated with the indicated concentrations of Ara-C. The samples were stratified into Ara-C-sensitive and Ara-C-resistant groups based on their IC50 values. **H**: Representative flow cytometry histograms (left) and quantitative analysis (right) of intracellular neutral lipids in AML samples, assessed by BODIPY 493/503 staining. **I**: The relative mRNA expression levels of STAT3, SREBF1, and CPT2 in the primary AML samples.*P < 0.05; **P < 0.01; two-tailed t test. Data are presented as mean ± SD and are representative of three independent experiments.

**Supplemental Tables**

**Table S1. gRNA sequences used for STAT3 knockout**

| Primer | Sequence (5’-3’) |
| --- | --- |
| Forward primer | CACCGCAGCTTGACACACGGTACC |
| Reverse primer | AAACGGTACCGTGTGTCAAGCTGC |

**Table S2. The sequences of human gene-specific primers**

| Primer | Sequence (5’-3’) |
| --- | --- |
| SREBP1 | F: ACAGTGACTTCCCTGGCCTAT |
|  | R: GCATGGACGGGTACATCTTCAA |
| SCD | F: TCTAGCTCCTATACCACCACCA |
|  | R: TCGTCTCCAACTTATCTCCTCC |
| ACSL3 | F: GGCGTAGCGGTTTTGACA |
|  | R: CCAGTCCTTCCCAACAACGA |
| FASN | F: AAGGACCTGTCTAGGTTTGATGC |
|  | R: TGGCTTCATAGGTGACTTCCA |
| SCD5 | F: ATGTCGTCCTGATGAGCTTG |
|  | R: CAGGAGGAAGCAGAAGTAGG |
| ACLY | F: GACTTCGGCAGAGGTAGAGC |
|  | R: TCAGGAGTGACCCGAGCATA |
| FADS1 | F: CAGACATCAACATGCATCCCT |
|  | R: AGAAGTATTTGTGCTGGTGGT |
| FADS2 | F: TTAACTTCCAGATTGAGCACCA |
|  | R: TTGGCACATAGAGACTTCACC |
| CPT2 | F: CATACAAGCTACATTTCGGGACC |
|  | R: AGCCCGGAGTGTCTTCAGAA |
| CPT1A | F: TCCAGTTGGCTTATCGTGGTG |
|  | R: TCCAGAGTCCGATTGATTTTTGC |
| ECH1 | F: ATAGTGGCTTCTCGCAGACTC |
|  | R: CAGTGAGGCGAAGGCTAATAC |
| ACAA1 | F: GCGGTTCTCAAGGACGTGAAT |
|  | R: GTCTCCGGGATGTCACTCAGA |
| ACOX3 | F: CTCGATGCCTACCGAGCAAG |
|  | R: TGGTTTTCTTAAAGCGGAGCA |
| ACSL4 | F: CATCCCTGGAGCAGATACTCT |
|  | R: TCACTTAGGATTTCCCTGGTCC |
| ALDH9A1 | F: GTCGCAGCCGCTCAATTAC |
|  | R: CCTTTGCATTTTGAACAGCCAA |
| HADHA | F: ATATGCCGCAATTTTACAGGGT |
|  | R: ACCTGCAATAAAGCAGCCTGG |
| ACOX1 | F: ACTCGCAGCCAGCGTTATG |
|  | R: AGGGTCAGCGATGCCAAAC |
| STAT3 | F: CTGCCCCATACCTGAAGACC |
|  | R: TCCTCACATGGGGGAGGTAG |
| β-actin | F: ACTCTTCCAGCCTTCCTTCC |
|  | R: CGTACAGGTCTTTGCGGATG |

**Table S3. Sequences of primers used for ChIP-qPCR**

| Primer | Sequence (5’-3’) |
| --- | --- |
| CPT2 promoter | F: CTGAGATGCCCTTTGTCCTCAC |
|  | R: GCTGCTCGTCTCCCTTCCCTTCC |
| SREBP1 promoter | F: GACCGTGAGTAACCTCGCAAG |
|  | R: GACTCTCCAGCCCTGTGATG |

**Table S4. Sequences of shRNAs and siRNAs**

| Primer | Sequence (5’-3’) |
| --- | --- |
| sh#1-STAT3 Forward | CCGGGCACAATCTACGAAGAATCAACTCGAGTT  GATTCTTCGTAGATTGTGCTTTTTG |
| sh#1-STAT3 Reverse | AATTCAAAAAGCACAATCTACGAAGAATCAAC  TCGAGTTGATTCTTCGTAGATTGTGC |
| sh#2-STAT3 Forward | CCGGGCAAAGAATCACATGCCACTTCTCGAGAA  GTGGCATGTGATTCTTTGCTTTTTG |
| sh#2-STAT3 Reverse | AATTCAAAAAGCAAAGAATCACATGCCACTTCT  CGAGAAGTGGCATGTGATTCTTTGC |
| sh#1-SREBP1 Forward | CCGGCGGAGAAGCTGCCTATCAACTCGAGTTGATAGGCAGCTTCTCCGTTTTTG |
| sh#1-SREBP1 Reverse | AATTCAAAAACGGAGAAGCTGCCTATCAACTCGAGTTGATAGGCAGCTTCTCCG |
| sh#2-SREBP1 Forward | CCGGCCACAACGCCATCGAGAAACTCGAGTTTCTCGATGGCGTTGTGGTTTTTG |
| sh#2-SREBP1 Reverse | AATTCAAAAACCACAACGCCATCGAGAAACTCGAGTTTCTCGATGGCGTTGTGG |
| siCPT2 | GACACUAUCACCUUCAAGA |

**Table S5. Preliminary Pharmacokinetic Parameters for W1307**

| Parameter | 10 mg/kg i.p. |
| --- | --- |
| T1/2 (hr) | 1.22 |
| Tmax (hr) | 0.250 |
| Cmax (ng/mL) | 4300 |
| AUC_0-t_ (hr*ng/mL) | 4800 |
| AUC_0-∞_ (hr*ng/mL) | 4815 |
| MRT_0-t_ (hr) | 1.01 |
| MRT_0-∞_ (hr) | 1.04 |

Values are the average of three runs. Cmax, maximum observed plasma concentration. Tmax, time to reach maximum plasma concentration. T1/2, terminal elimination half-life. AUC_0-t_, area under the plasma concentration-time curve from time zero to the last measurable time point. AUC_0-∞_, area under the plasma concentration-time curve from time zero extrapolated to infinity. MRT_0-t_, mean residence time from time zero to the last measurable time point; MRT_0-∞_, mean residence time from time zero extrapolated to infinity.
